# Home-Based Respiratory Physiotherapy and Telephone-Based Psychological Support for COVID-19 Survivors in Peru: Protocol for a Randomized Controlled Trial

**DOI:** 10.2196/36001

**Published:** 2022-10-24

**Authors:** Anderson N Soriano-Moreno, Elaine C Flores, Stella M Hartinger, Claudia Y Mallma, Arnold A Diaz, Gonzalo E Gianella, Juan A Galvez-Buccollini, Abdiel H Coico-Lama, German Malaga, Eufemia Fajardo, Rubí Paredes-Angeles, Sharlyn Otazú-Alfaro, Andres G Lescano, William Checkley, WAYRA Trial Investigators

**Affiliations:** 1 Pulmonary Training Program in Peru Universidad Peruana Cayetano Heredia Lima Peru; 2 Center for Global Non-Communicable Disease Research and Training Johns Hopkins University Baltimore, MD United States; 3 Clima, Latin American Center of Excellence on Climate Change and Health Universidad Peruana Cayetano Heredia Lima Peru; 4 Centre on Climate Change and Planetary Health London School of Hygiene and Tropical Medicine London United Kingdom; 5 Center for Innovation in Global Health Stanford University Palo Alto, CA United States; 6 Escuela Profesional de Tecnología Médica Universidad Nacional Mayor de San Marcos Lima Peru; 7 Department of Physical Medicine and Rehabilitation Hospital EsSalud Alberto Sabogal Sologuren Lima Peru; 8 Emerge, Climate Change and Emerging Disease Research Unit Universidad Peruana Cayetano Heredia Lima Peru; 9 CRONICAS Center of Excellence in Chronic Diseases Universidad Peruana Cayetano Heredia Lima Peru; 10 School of Medicine Universidad Peruana Cayetano Heredia Lima Peru; 11 Department of Psychiatry Clínica Anglo Americana Lima Peru; 12 Clinical and Epidemiological Research Unit School of Medicine Universidad Peruana Unión Lima Peru; 13 Department of Medicine Hospital Nacional Cayetano Heredia Lima Peru; 14 Department of Physical Medicine and Rehabilitation Hospital Nacional Cayetano Heredia Lima Peru; 15 Grupo de Estudios Avances en Medición Psicológica Universidad Nacional Mayor de San Marcos Lima Peru; 16 Mental Health Research Unit Instituto Peruano de Orientación Psicológica Lima Peru; 17 Division of Pulmonary and Critical Care School of Medicine Johns Hopkins University Baltimore, MD United States

**Keywords:** COVID-19, pulmonary rehabilitation, psychiatric rehabilitation, mental health, clinical trial

## Abstract

**Background:**

Both pulmonary and mental health are affected following hospitalization for COVID-19 pneumonia. Pulmonary rehabilitation therapy has demonstrated benefits in improving mental health, but no validated combined programs that include mental health have been proposed.

**Objective:**

This article presents the design of a trial that aimed to assess whether the participation in a combined rehabilitation program that includes home-based respiratory physiotherapy and telephone-based psychological support is associated with a greater improvement of pulmonary and mental health outcomes 7-12 weeks after COVID-19 hospitalization discharge compared with posthospital usual care provided by a public Peruvian hospital.

**Methods:**

WAYRA (the word for air in the Quechua language) was an open-label, unblinded, two-arm randomized controlled trial. We recruited 108 participants aged 18-75 years who were discharged from the hospital after COVID-19 pneumonia that required >6 liters/minute of supplemental oxygen during treatment. Participants were randomly assigned at a 1:1 ratio to receive the combined rehabilitation program or usual posthospital care provided by a public Peruvian hospital. The intervention consisted of 12 at-home respiratory rehabilitation sessions and 6 telephone-based psychological sessions. The primary outcome was the 6-minute walk distance. Secondary outcomes included lung function, mental health status (depression, anxiety, and trauma), and quality of life. Outcomes were assessed at baseline (before randomization) and at 7 and 12 weeks after hospital discharge to assess the difference between arms.

**Results:**

This study was funded by the Peruvian National Council of Science Technology and Technology Innovation in July 2020. Ethics approval was obtained on September 2, 2020. Recruitment and data collection occurred between October 2020 and June 2021. Results are expected to be published by the end of 2022.

**Conclusions:**

WAYRA was the first randomized controlled trial evaluating combined pulmonary-mental health rehabilitation for hospitalized COVID-19 survivors in resource-limited settings, potentially providing a foundation for the cost-effective scale-up of similar multidisciplinary rehabilitation programs.

**Trial Registration:**

ClinicalTrials.gov NCT04649736; https://clinicaltrials.gov/ct2/show/NCT04649736

**International Registered Report Identifier (IRRID):**

RR1-10.2196/36001

## Introduction

The COVID-19 pandemic is resulting in millions of survivors with long-term complications [1]. Complications are more frequent in patients who required hospitalization, affecting both physical and mental health [1]. Systematic reviews show that 24% to 36% and 40% to 58% of patients with COVID-19 report dyspnea and fatigue after acute illness, respectively [2,3]. The frequency of depression and anxiety in COVID-19 survivors ranges from 12% to 15% and 13% to 22%, respectively [2-5].

Although recommendations for post-COVID-19 rehabilitation already exist [6-9], the evidence is scarce from different aspects. First, most studies have focused on pulmonary rehabilitation [10-12], but no study has evaluated a rehabilitation program that integrates a psychological component. The high frequency of mental illness suggests that patients may benefit from physiological support. Second, no published clinical trial has evaluated a home-based rehabilitation program, an approach that could have advantages over inpatient programs and comparable efficacy [12]. After hospitalization, COVID-19 patients could have several health limitations that make it difficult to visit health care facilities for rehabilitation. Third, most strategies to address the sequelae of COVID-19 have been developed in high-income countries and therefore do not consider some of the challenges specific to developing countries, such as the availability and quality of professionals and infrastructure to carry out rehabilitation [13].

Considering that COVID-19 is a disease that results in short-, medium-, and long-term physical and mental impairment, and that limited rehabilitation strategies have been evaluated, this protocol describes the design and methodology of a randomized controlled trial that aimed to evaluate the effects of integrated home-based respiratory physiotherapy combined with a telephone-based psychological intervention on pulmonary and mental health–related outcomes at 7 and 12 weeks after hospital discharge in patients with COVID-19 pneumonia. The primary objective of the trial was to compare 6-minute walking distances at 7 and 12 weeks after hospital discharge among participants in a combined program versus those who received usual posthospital care provided by a Peruvian public hospital.

## Methods

### Study Design

**Figure 1:**
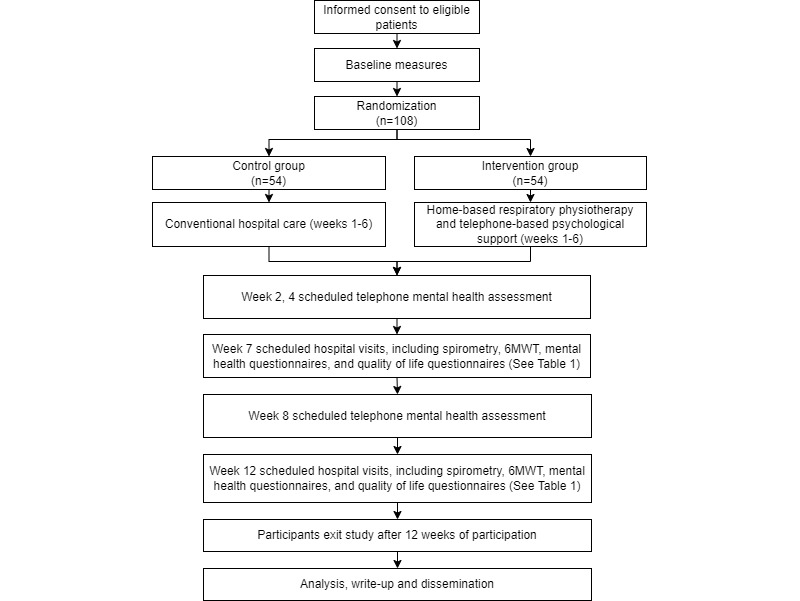
Expected study enrollment and timeline diagram. 6MWT: 6-minute walk test.

WAYRA (the word for air in the Quechua language) was a randomized, controlled, open-label, two-arm clinical trial that evaluated the efficacy of a 6-week home-based respiratory physiotherapy and telephone-based psychological program compared to usual posthospital care provided by a public Peruvian hospital, aimed at improving exercise tolerance, lung function, mental health, and quality of life (QoL) outcomes. A baseline assessment was conducted at hospital discharge, with two follow-ups at week 7 and 12 (Figure 1). Due to the nature of the rehabilitation interventions, blinding of participants and the researchers was not possible; however, the data scientist conducting the data analysis was blinded. The trial was registered at ClinicalTrials.gov (NCT04649736).

### Study Setting and Population

This study took place in the COVID-19 ward at Hospital Nacional Cayetano Heredia (HNCH), a public tertiary-care hospital and a major referral center that serves approximately 3 million people from underserved neighborhoods in the northern metropolitan area of Lima, the capital of Peru. HNCH has been one of the main COVID-19 national referral centers throughout the pandemic, with 241 hospitalization beds and 23 intensive care unit beds [14]. During the pandemic, more than half of Lima’s population reported depressive symptoms, with increased rates of symptoms reported among young people with low income and without higher education [15].

The study population consisted of confirmed cases of COVID-19, diagnosed by serological or molecular tests, including members of both sexes, between 18 and 75 years of age, discharged from hospitalization, who received high-flow oxygen (>6 liters/minute) treatment at any point during hospitalization. Participants with prior respiratory pathology or psychiatric diagnoses were excluded. The complete eligibility criteria are provided in Textbox 1.

**Inclusion and exclusion criteria.**

**Inclusion criteria**

- Age between 18 and 75 years
- Discharged from hospitalization with a confirmed COVID-19 diagnosis
- Capable of understanding study procedures
- Capable of providing informed consent
- Required oxygen flow >6 liters/minute or through a high-flow device at any time during hospitalization
- Evaluated by the rehabilitation service at least once during hospitalization

**Exclusion criteria**

- Contraindications to the 6-minute walk test
- Contraindications to spirometry
- Complications during the baseline 6-minute walk test
- Neurological pathology, neuropathy, limb dysfunction, or other underlying physical disability that makes physical exercise impossible
- Pregnant or breastfeeding
- No access to the internet or a telephone line
- History or existing lung diseases such as asthma, chronic obstructive pulmonary disease, fibrosis, or tuberculosis
- Moderate or severe heart disease (Grade III or IV, New York Heart Association)
- Diagnosed with another severe disease in the last 6 months
- Severe depression or suicidal ideation
- Taking any medication for depression and/or anxiety, or another medication prescribed by a psychiatrist before the onset of COVID-19
- Cognitive impairment or sensory disturbance

### Enrollment

A trained nurse made the initial contact and prescreening, starting with a daily review of the list of hospitalized patients during the enrollment period, to identify potentially eligible participants among newly discharged patients or those close to discharge in the following days. Potential participants were informed about the study and its procedures and were invited to participate. Those who accepted received a detailed explanation of the nature of the study, randomization, study procedures, their potential risks and benefits, their rights as participants, and the study timelines. Potential subjects were explicitly told that participation was not mandatory, that there was no penalty for refusing to participate, and that their clinical treatment at the hospital would not be compromised in any way if they refuse to participate or opt out of the study at any time. All interested patients received the screening informed consent form (Multimedia Appendix 1) for their review, and they provided a signature once all their questions and concerns were addressed.

After obtaining participant consent and signature, a trained nurse performed the screening assessments, including review of eligibility criteria compliance and baseline measurements. These included cognitive status (assessed using the Montreal Cognitive Assessment, visual impairment version [MoCA-BLIND] [16]), sociodemographic and clinical data, baseline respiratory symptoms (using the St. George’s Respiratory Questionnaire [SGRQ]), depressive symptoms (using the Nine-Item Patient Health Questionnaire [PHQ-9]), anxiety symptoms (using the Generalized Anxiety Disorder Assessment [GAD-7]), posttraumatic stress disorder symptoms (Impact of Event Scale-Revised [IES-R]), and QoL (using the 36-Item Short Form Survey [SF-36] and EuroQuol-5D [EQ-5D]). Subsequently, a trained physician assessed the participants’ exercise tolerance with the 6-minute walk test (6MWT) and pulmonary function with a spirometry test. Participants were screened for severe depressive symptoms. Those with a GAD-7 score ≥15 or IES-R score ≥37 were referred to a psychiatrist for evaluation and treatment. Participants with a PHQ-9 score ≥20 and self-reported suicidal ideation during the psychological evaluation were immediately referred to mental health services for treatment and were excluded from the study. Patients who completed the baseline assessments and met eligibility criteria were enrolled into the study. The recruitment team communicated with the study coordinator who performed a baseline data quality verification, the allocation to one of two arms (control or intervention), and assigned a participant ID code.

### Randomization

We used a blinded list preuploaded in REDcap [17], which was created by a data administrator who was not involved in study recruitment. To achieve balance in the number of participants assigned to each study arm, the treatment arm was randomized at a 1:1 ratio using permuted blocks of variable size, randomly varying from 2, 4, and 6 participants. The blocks were randomized using the Stata v16.0 *ralloc* command (StataCorp). Finally, all participants were scheduled for follow-up visits as described in Table 1. Sample selection and enrollment took place between the day before hospital discharge and up to 3 days after discharge.

**Table 1:** Data collection schedule.

Time point				Study period (week)																											
				Enrollment (–1)		Baseline (0)		Postallocation																							Close-out (12)
								1		2		3		4		5		6		7		8		9		10		11			
**Enrollment**																															
	Informed consent		✓																												
	Eligibility screen		✓																												
	Allocation				✓																										
**Interventions**																															
	**Intervention arm**																														
		Home-based respiratory rehabilitation					✓		✓		✓		✓		✓		✓														
		Telephone psychological support					✓		✓		✓		✓		✓		✓														
	Control arm: conventional care						✓		✓		✓		✓		✓		✓		✓		✓		✓		✓		✓		✓		
**Assessments**																															
	Sociodemographic questionnaire		✓																												
	Primary outcome: 6MWT^a^ distance				✓														✓										✓		
	**Secondary outcomes**																														
		Spirometry^b^			✓														✓										✓		
		Respiratory symptoms (SGRQ^c^)			✓														✓										✓		
		Quality of life (SF-36^d^, EQ-5D^e^)			✓														✓										✓		
		Mental health (PHQ-9^f^, GAD-7^g^, IES-R^h^)			✓				✓				✓						✓		✓								✓		
		Cognitive function (MoCA-BLIND^i,^ visual impairment)			✓				✓				✓						✓		✓								✓		

^a^6MWT: 6-minute walk test.

^b^Includes forced expiratory volume in 1 second (FEV1), forced vital capacity (FVC), and FEV1/FVC ratio.

^c^SGRQ: Saint George’s Respiratory Questionnaire.

^d^SF-36: 36-Item Short Form Survey.

^e^EQ-5D: EuroQuol 5D.

^f^PHQ-9: Nine-Item Patient Health Questionnaire.

^g^GAD-7: Generalized Anxiety and Depression.

^h^IES-R: Impact of Event Scale-Revised.

^i^MoCA-BLIND: Montreal Cognitive Assessment visual impairment version.

### Comparison Groups

#### Intervention Arm

The intervention consisted of 12 sessions of home-based respiratory rehabilitation and 6 sessions of weekly psychological support delivered through telephone home calls throughout a period of 6 weeks. Participants began the rehabilitation program no later than 1 week after hospital discharge.

Respiratory rehabilitation consisted of 12 sessions (1-3 per week depending on the participant’s availability) performed at home under trained physiotherapist guidance. Each respiratory rehabilitation session lasted approximately 1 hour. In the first rehabilitation session, the physiotherapist brought the necessary equipment to perform the exercises (1-kilogram abdominal weight and a small rubber ball). In each session, the participant performed the following exercises: respiratory muscle training by breathing through pursed lips and breathing education exercises, coughing exercises to produce effective coughs, diaphragm training by teaching diaphragmatic contractions in the supine position with a light weight placed on the anterior abdominal wall, and stretching exercises whereby the participant was placed in a supine or lateral position with the knees bent to correct for lumbar lordosis. In addition, participants performed arm movements in flexion, horizontal extension, abduction, and external rotation. Participants were instructed to perform pursed-lip breathing and effective coughing on the days they did not receive assisted therapy, with a frequency of 30 times per day. The day of nonattendance at respiratory therapy was recorded. Given the risk of exposure to COVID-19, physiotherapists followed a strict biosafety protocol involving the use of personal protective equipment and a thorough disinfection process at each visit. In addition, rehabilitation was ideally performed in a well-ventilated room and the participant wore a surgical mask during visits as an extra protective measure.

The psychological support intervention consisted of an introductory session (Session 0) and 6 1-hour structured sessions based on emotion-centered problem-solving therapy (EC-PST). A trained licensed psychologist provided these sessions once a week by telephone home calls following the guidelines of the Inter-Agency Standing Committee (IASC) on Mental Health and Psychosocial Support in Humanitarian and Disaster Emergencies of the World Health Organization [18]. The EC-PST is an update of the classic problem-solving therapy that emphasizes guiding patients to better understand and manage their emotional reactions to stressful events as a means of coping with the negative effects of these stressors [19]. EC-PST reviews four components: (1) Planful Problem Solving, (2) Overcoming Brain Overload, (3) Enhancing Motivation for Action, and (4) Stop and Slow Down. In addition, we provided each participant with a printed workbook that included the themes and topics that were developed in each session, and the psychologist recorded the main points of the session using an adhoc evaluation form. Table 2 summarizes the objectives of each session.

**Table 2:** Psychological support objectives.

Session	Content	Objectives
0	Introductory session and psychological history	Take the participant’s psychological medical history; evaluate stressful events, consequences, and the participant’s perception of what they are experiencing, through the functional behavioral analysis
1	Psychoeducation session	Provide information on the types of problem orientation and problem-solving styles; identify the problem-solving style employed by the patient
2	Toolbox training 1 “Stop and Slow Down”	Teach the “Stop and Slow Down” tool to better modulate emotional reactions to stressful stimuli. Strategies included are counting, guided imagery or visualization, deep breathing, “fake” yawning, conscious meditation, relaxation exercises, deep-muscle exercise or conscious walking, talking to someone, prayer, and the DAPA^a^ Method for Emotional Regulation
3	Toolbox training 2 “Overcome Brain Overload” and “Boost Motivation to Act”	Teach “Beat Brain Overload” tools to overcome barriers or obstacles to effective problem-solving, particularly when under stress, through three specific strategies: externalization, visualization, and simplification; orienting people to overcome low motivation and feelings of hopelessness through use of the “Power Motivation to Act” tool through externalization and visualization
4	Toolbox training 3 “Plan the Solution to Your Problems”	Explain and teach the four effective problem-solving steps: define the problem, generate solution alternatives, make the decision, and implement and verify the solution
5	Guided practice	Assist participants in adjusting the problem-solving skills they have acquired; monitor the application of these principles; help participants meaningfully integrate the various tools; reinforce the participant’s progress as a means to further increase the sense of self-efficacy
6	Prognosis assessment and termination of the psychological therapy	Prognosis assessment: discuss possible difficulties that the participant may have to help them plan their actions; evaluate the possibility of using the tools taught in the possibility of managing these difficulties. Therapy termination: review the EC-PST^b^ objectives established in the initial sessions; ask the patient for examples of how these objectives have been met

^a^DAPA: Dementia and Physical Activity: stop, walk slowly, think, act.

^b^EC-PST: emotion-centered problem-solving therapy.

#### Control Arm

Participants assigned to the control arm received posthospital usual care delivered by a Peruvian public hospital consisting of discharge recommendations and a telephone follow-up for 14 days provided by the same hospital. Although there are no national guidelines for standardized discharge indications in Peru, many hospitals recommended patients to perform home breathing exercises and symptomatic respiratory treatment. There are currently no diagnostic or follow-up plans for patients that experience or develop mental illness or a strategy for respiratory or psychological rehabilitation in most hospitals in Peru. We collected data about any additional nonstudy therapies that participants may have received from any internal or external health care provider.

### Measured Outcomes

#### Primary Outcome

The primary health outcome was the change in 6-minute walk distance (6MWD) at 7 and 12 weeks after discharge compared to baseline. The 6MWT is a submaximal effort test of cardiorespiratory functional capacity with correlation to QoL and independence in activities of daily living [20]. The 6MWT is widely used for the follow-up of patients after hospitalization because patients can self-regulate the intensity of the activity, resting as often as desired, leading to minimal risks of adverse events during the activity [20]. The test was performed in a 20-meter hospital aisle marked according to the recommendations of the American Thoracic Society [21,22]. Participants who demonstrated instability while standing, generalized weakness, or those with a decrease in oxygen saturation below 90% or increased heart rate of >130 beats per minute while standing did not perform the test. In this case, the participants were considered to have covered 0 meters. Each participant only attempted the test once. The number of meters walked in the 6MWT was calculated by the assessor and recorded in the data collection form.

#### Secondary Outcomes

A trained physician measured forced expiratory volume in the first second (FEV1), forced vital capacity (FVC), and the FEV1/FVC ratio using a portable spirometer (Easy-On-PC). These spirometers are frequently used in pulmonary research because their calibration remains stable over time [23]. We aimed to obtain at least three acceptable and two reproducible tests following joint recommendations of the American Thoracic Society and European Respiratory Societies [24] or until the participant was no longer able to tolerate the procedure. Results were recorded on a secure interface (EasyOnConnect) on a personal computer.

For mental health and QoL assessments, participants responded to questionnaires administered by a trained health worker. We used PHQ-9, GAD-7, and IES-R questionnaires to assess mental health, including depression [25], anxiety [26], and posttraumatic stress disorder symptoms [27], respectively. Mental health questionnaires were administered at 2, 4, 7, 8, and 12 weeks after hospitalization by telephone home calls. We used the SF-36 v2 [28] and EQ-5D [29] questionnaires to measure QoL at 7 and 12 weeks after discharge. The psychometric properties of the SF-36 questionnaire have been widely studied and consist of 36 items assessing 8 dimensions: physical functioning, physical role, bodily pain, general health, vitality, social functioning, emotional role, and mental health [30]. The details of the measurements for the primary and secondary outcomes are summarized in Table 3. We used the SGRQ to assess respiratory symptoms, an instrument recommended to measure changes in respiratory health following interventions [31]. We measured respiratory symptoms at 7 and 12 weeks after hospital discharge (Table 1).

**Table 3:** Outcomes and instruments in the WAYRA study.

Outcome	Instruments
Pulmonary function	6-minute walk test, spirometry [20]
Respiratory symptoms	St. George’s Respiratory Questionnaire (SGRQ) [31]
Depressive symptoms	Patient Health Questionnaire (PHQ-9) [25]
Anxiety symptoms	Generalized Anxiety Disorder (GAD-7) [26]
Posttraumatic stress disorder	Impact Event Scale-Revised (IES-R) [27]
Quality of life	36-Item Short-Form Health Survey [28], EuroQol-5D (EQ-5D) [29]

### Statistical Analyses

We will perform an intention-to-treat analysis of primary and secondary outcomes at 7 and 12 weeks after hospital discharge. The final analysis will be performed when all 108 participants completed the trial. We will compare the 6MWD (meters), FVC (liters), FEV1 (liters), and questionnaire scores at 7 and 12 weeks after hospitalization discharge between the intervention and control arms. In the secondary analyses, we will compare the proportion of participants with respect to restrictive spirometry pattern, depression, anxiety, and posttraumatic stress disorder at 7 and 12 weeks after hospital discharge between the intervention and control arms. In addition, following the recommendation by Cocks and Torgerson [32], we will use a one-sided 80% CI to determine if this trial should proceed to a phase III trial. As a sensitivity analysis, the association between the number of therapies received and outcomes will be examined, adjusting for potential confounders that will be identified by a causal acyclic diagram. There will be no formal interim analysis of the data.

### Sample Size Calculation

We used STATA Version 16 (StataCorp) to calculate the ideal sample size. We attempted to enroll 108 participants in total, allocating 54 to each treatment arm. We estimate that a sample size of 86 participants would be sufficient to detect a mean difference between arms of 55.1 meters in the 6MWT according to the study by Liu et al [11] considering a 95% CI and 90% power. Assuming a potential 20% rate of rejection or loss to follow-up, we aimed to achieve a sample of 108 participants.

### Ethics Approval

Ethical approval of the study protocol and informed consent form was obtained from the Institutional Review Board at Universidad Peruana Cayetano Heredia (#202852) and HNCH (#085-2020). Likewise, any changes in the protocol were reported to the mentioned ethics committees. Written informed consent was requested from all participants prior to any data collection. To protect participants’ confidentiality, all collected data of clinical assessments were linked to a unique ID code. Data were registered in paper form and electronically. The electronic information was stored, backed-up, and secured by password protection in the RedCap server. Paper forms were archived in secure locked cabinets in the HNCH. All confidential information, including participants’ contact details or other sensitive data, was only accessible to authorized staff from the project. Long-term data management complied with Universidad Peruana Cayetano Heredia research policy. The Peruvian National Health Institute reviewed the protocol and determined that it was exempt from review by the Ethics Committee due to the nonpharmacological nature of the intervention.

### Benefits and Risk of Participation

Both control- and intervention-arm participants benefited from the study. These benefits included education and counseling on respiratory disease risk factors, reporting of pulmonary function test results by a specialist physician, and referral to trained personnel if necessary.

Potential risks of respiratory rehabilitation were oxygen desaturation, palpitations, sweating, arrhythmias, chest tightness, shortness of breath, and muscle aches. Potential risks of psychological support were symptom substitution, dependence on the physiotherapist, stigmatization, problems in social relationships or even separation, as well as alcohol or drug abuse, deliberate self-harm, and suicidal ideation or attempts. Adverse events of the interventions were collected after participants provided informed consent and were duly enrolled in the study. If adverse events were detected, the field staff immediately notified the study coordinator and the management protocol was activated.

## Results

The trial, which obtained funding in July 2020, was approved by the Institutional Review Board on September 2, 2020. Data collection began in October 2020. Enrollment of participants began in October 2020 and was completed in June 2021. Results of the study are expected to be published by the end of 2022.

## Discussion

### Summary

WAYRA is the first randomized controlled trial evaluating integrated pulmonary and mental health rehabilitation for hospitalized COVID-19 survivors in a low- and middle-income country setting. Through this trial, we gained experience in implementing a remote rehabilitation program in a resource-limited country and will generate evidence on pulmonary, mental health, and QoL alterations following COVID-19 the first 3 months after hospitalization. We hypothesize that participation in the combined program will improve pulmonary, physical, and mental health outcomes. We hope that our study will provide a reliable basis for further clinical trials focused on comprehensive rehabilitation in patients hospitalized for severe COVID-19 and other lung diseases.

The burden of long-term complications of COVID-19 will increase because, although several countries have achieved broad vaccination coverage, newly emerging variants may be resistant to vaccines [33]. The most frequently affected systems are respiratory and physical, although psychological symptoms are also common [3]. Despite this, there are still few published studies that have proposed strategies for COVID-19 survivors with long-term complications and far fewer that include mental health support. There are approximately 200 trials registered with ClinicalTrials.gov evaluating rehabilitation programs for COVID-19 survivors; most include pulmonary rehabilitation, whereas only a few have a psychological component [34].

### Strengths and Limitations

The main strength of this study is that the calculated sample size has the power to detect a clinically significant difference between the groups. Nevertheless, this study has some limitations to be considered. First, mental health and QoL outcomes will be assessed with screening tools. Second, this trial has an open-label design due the nature of the intervention.

### Conclusions

In conclusion, the COVID-19 pandemic will result in millions of people prone to develop respiratory and mental health sequelae worldwide, and health systems are not prepared to respond adequately to this situation. Little evidence exists on rehabilitation strategies, and most of the existing evidence does not come from low- and middle-income countries that face additional challenges compared with developed countries. The clinical trial described in this protocol will generate useful evidence to develop effective interventions to benefit patients after COVID-19, and will serve as a basis to help plan future studies evaluating pulmonary rehabilitation strategies and mental psychological support.

## Appendix

Multimedia Appendix 1 Informed consent form.

Multimedia Appendix 2 CONSORT-eHEALTH v1.6.1

## Abbreviations

6MWT: 6-minute walk test
6MWD: 6-minute walk distance
EC-PST: emotion-centered problem-solving therapy
EQ-5D: EuroQol-5D
FEV1: forced expiratory volume in the first second.
FVC: forced vital capacity.
GAD-7: Generalized Anxiety Disorder
HNCH: Hospital Nacional Cayetano Heredia
IASC: Inter-Agency Standing Committee
IES-R: Impact Event Scale-Revised
MoCA-BLIND: Montreal Cognitive Assessment, visual impairment version
PHQ-9: Patient Health Questionnaire
QoL: quality of life
SF-36: 36-Item Short Form health survey
SGRQ: St. George’s Respiratory Questionnaire

## References

[1] Chen C, Haupert SR, Zimmermann L, Shi X, Fritsche LG, Mukherjee B (2022). Global prevalence of post COVID-19 condition or long COVID: a meta-analysis and systematic review. J Infect Dis.

[2] Lopez-Leon S, Wegman-Ostrosky T, Perelman C, Sepulveda R, Rebolledo P, Cuapio A, Villapol S (2021). More than 50 long-term effects of COVID-19: a systematic review and meta-analysis. Sci Rep.

[3] Nasserie T, Hittle M, Goodman SN (2021). Assessment of the frequency and variety of persistent symptoms among patients with COVID-19: a systematic review. JAMA Netw Open.

[4] Michelen M, Manoharan L, Elkheir N, Cheng V, Dagens A, Hastie C, O'Hara M, Suett J, Dahmash D, Bugaeva P, Rigby I, Munblit D, Harriss E, Burls A, Foote C, Scott J, Carson G, Olliaro P, Sigfrid L, Stavropoulou C (2021). Characterising long COVID: a living systematic review. BMJ Glob Health.

[5] Almas T, Malik J, Alsubai AK, Jawad Zaidi SM, Iqbal R, Khan K, Ali M, Ishaq U, Alsufyani M, Hadeed S, Alsufyani R, Ahmed R, Thakur T, Huang H, Antony I, Antony I, Bhullar A, Kotait F, Al-Ani L (2022). Post-acute COVID-19 syndrome and its prolonged effects: an updated systematic review. Ann Med Surg.

[6] (2021). Living guidance for clinical management of COVID-19. World Health Organization.

[7] Maley JH, Alba GA, Barry JT, Bartels MN, Fleming TK, Oleson CV, Rydberg L, Sampsel S, Silver JK, Sipes S, Verduzco-Gutierrez M, Wood J, Zibrak JD, Whiteson J (2022). Multi-disciplinary collaborative consensus guidance statement on the assessment and treatment of breathing discomfort and respiratory sequelae in patients with post-acute sequelae of SARS-CoV-2 infection (PASC). PM R.

[8] Fine JS, Ambrose AF, Didehbani N, Fleming TK, Glashan L, Longo M, Merlino A, Ng R, Nora GJ, Rolin S, Silver JK, Terzic CM, Verduzco-Gutierrez M, Sampsel S (2022). Multi-disciplinary collaborative consensus guidance statement on the assessment and treatment of cognitive symptoms in patients with post-acute sequelae of SARS-CoV-2 infection (PASC). PM R.

[9] Webber SC, Tittlemier BJ, Loewen HJ (2021). Apparent discordance between the epidemiology of COVID-19 and recommended outcomes and treatments: a scoping review. Phys Ther.

[10] Yang L, Yang T (2020). Pulmonary rehabilitation for patients with coronavirus disease 2019 (COVID-19). Chronic Dis Transl Med.

[11] Liu K, Zhang W, Yang Y, Zhang J, Li Y, Chen Y (2020). Respiratory rehabilitation in elderly patients with COVID-19: a randomized controlled study. Complement Ther Clin Pract.

[12] Fugazzaro S, Contri A, Esseroukh O, Kaleci S, Croci S, Massari M, Facciolongo NC, Besutti G, Iori M, Salvarani C, Costi S, Reggio Emilia COVID-19 Working Group (2022). Rehabilitation interventions for post-acute COVID-19 syndrome: a systematic review. Int J Environ Res Public Health.

[13] Bickton FM, Shannon H (2022). Barriers and enablers to pulmonary rehabilitation in low- and middle-income countries: a qualitative study of healthcare professionals. Int J Chron Obstruct Pulmon Dis.

[14] Mejía F, Medina C, Cornejo E, Morello E, Vásquez S, Alave J, Schwalb A, Málaga G (2020). Oxygen saturation as a predictor of mortality in hospitalized adult patients with COVID-19 in a public hospital in Lima, Peru. PLoS One.

[15] Antiporta DA, Cutipé YL, Mendoza M, Celentano DD, Stuart EA, Bruni A (2021). Depressive symptoms among Peruvian adult residents amidst a National Lockdown during the COVID-19 pandemic. BMC Psychiatry.

[16] Dawes P, Pye A, Reeves D, Yeung WK, Sheikh S, Thodi C, Charalambous AP, Gallant K, Nasreddine Z, Leroi I (2019). Protocol for the development of versions of the Montreal Cognitive Assessment (MoCA) for people with hearing or vision impairment. BMJ Open.

[17] Harris PA, Taylor R, Thielke R, Payne J, Gonzalez N, Conde JG (2009). Research electronic data capture (REDCap)--a metadata-driven methodology and workflow process for providing translational research informatics support. J Biomed Inform.

[18] (2007). IASC Guidelines on mental health and psychosocial support in emergency settings. Inter-Agency Standing Committee.

[19] Cuijpers P, de Wit L, Kleiboer A, Karyotaki E, Ebert DD (2018). Problem-solving therapy for adult depression: an updated meta-analysis. Eur Psychiatry.

[20] Diniz LS, Neves VR, Starke AC, Barbosa MP, Britto RR, Ribeiro AL (2017). Safety of early performance of the six-minute walk test following acute myocardial infarction: a cross-sectional study. Braz J Phys Ther.

[21] Veloso-Guedes C, Rosalen S, Thobias C, Andreotti R, Galhardo F, Oliveira da Silva A, Araujo O, Boin I (2011). Validation of 20-meter corridor for the 6-minute walk test in men on liver transplantation waiting list. Transplant Proc.

[22] ATS Committee on Proficiency Standards for Clinical Pulmonary Function Laboratories (2002). ATS statement: guidelines for the six-minute walk test. Am J Respir Crit Care Med.

[23] Skloot GS, Edwards NT, Enright PL (2010). Four-year calibration stability of the EasyOne portable spirometer. Respir Care.

[24] Graham BL, Steenbruggen I, Miller MR, Barjaktarevic IZ, Cooper BG, Hall GL, Hallstrand TS, Kaminsky DA, McCarthy K, McCormack MC, Oropez CE, Rosenfeld M, Stanojevic S, Swanney MP, Thompson BR (2019). Standardization of Spirometry 2019 Update. An Official American Thoracic Society and European Respiratory Society Technical Statement. Am J Respir Crit Care Med.

[25] Villarreal-Zegarra D, Copez-Lonzoy A, Bernabé-Ortiz A, Melendez-Torres GJ, Bazo-Alvarez JC (2019). Valid group comparisons can be made with the Patient Health Questionnaire (PHQ-9): a measurement invariance study across groups by demographic characteristics. PLoS One.

[26] García-Campayo J, Zamorano E, Ruiz MA, Pardo A, Pérez-Páramo M, López-Gómez V, Freire O, Rejas J (2010). Cultural adaptation into Spanish of the generalized anxiety disorder-7 (GAD-7) scale as a screening tool. Health Qual Life Outcomes.

[27] Gargurevich R, Luyten P, Fils J, Corveleyn J (2009). Factor structure of the Impact of Event Scale-Revised in two different Peruvian samples. Depress Anxiety.

[28] Alonso J, Prieto L, Antó JM (1995). The Spanish version of the SF-36 Health Survey (the SF-36 health questionnaire): an instrument for measuring clinical results. Med Clin.

[29] Konerding U, Elkhuizen SG, Faubel R, Forte P, Malmström T, Pavi E, Janssen MB (2014). The validity of the EQ-5D-3L items: an investigation with type 2 diabetes patients from six European countries. Health Qual Life Outcomes.

[30] Vilagut G, Ferrer M, Rajmil L, Rebollo P, Permanyer-Miralda G, Quintana J, Santed R, Valderas J, Domingo-Salvany A, Alonso J (2005). El Cuestionario de Salud SF-36 español: una década de experiencia y nuevos desarrollos. Gac Sanit.

[31] Jones PW (2005). St. George's Respiratory Questionnaire: MCID. COPD.

[32] Cocks K, Torgerson DJ (2013). Sample size calculations for pilot randomized trials: a confidence interval approach. J Clin Epidemiol.

[33] Wang Q, Guo Y, Iketani S, Nair MS, Li Z, Mohri H, Wang M, Yu J, Bowen AD, Chang JY, Shah JG, Nguyen N, Chen Z, Meyers K, Yin MT, Sobieszczyk ME, Sheng Z, Huang Y, Liu L, Ho DD (2022). Antibody evasion by SARS-CoV-2 Omicron subvariants BA.2.12.1, BA.4 and BA.5. Nature.

[34] McGregor G, Sandhu H, Bruce J, Sheehan B, McWilliams D, Yeung J, Jones C, Lara B, Smith J, Ji C, Fairbrother E, Ennis S, Heine P, Alleyne S, Guck J, Padfield E, Potter R, Mason J, Lall R, Seers K, Underwood M (2021). Rehabilitation Exercise and psycholoGical support After covid-19 InfectioN' (REGAIN): a structured summary of a study protocol for a randomised controlled trial. Trials.

