# Supplementary material for: Home-Based Respiratory Physiotherapy and Telephone-Based Psychological Support for COVID-19 Survivors in Peru: Protocol for a Randomized Controlled Trial
Source: JMIR Res Protoc. 2022 Oct 24;11(10):e36001. doi: 10.2196/36001 (PMC9595592; doi:10.2196/36001)
Supplement: Multimedia Appendix 1 [file resprot_v11i10e36001_app1.pdf]

CONSENTIMIENTO INFORMADO PARA PARTICIPAR EN UN ESTUDIO DE INVESTIGACIÓN – PRESELECCIÓN

INSTITUCIONES: **Universidad Peruana Cayetano Heredia (Lima, Perú)**

Johns Hopkins University (Baltimore, Maryland US)

The London School of Hygiene & Tropical Medicine

INVESTIGADOR: **Stella Maria Hartinger Peña, PhD**
E-MAIL: **stella.hartinger.p@upch.pe**

ESTUDIO: **Prevalencia de alteración de la función pulmonar post infección por COVID-19 e impacto de la participación en un programa piloto de rehabilitación integral a corto y mediano plazo.**

Lo invitamos a participar en un estudio de investigación que busca evaluar cuál es el daño que el COVID-19 puede dejar en los pulmones y en la salud mental. También busca ver si un programa de rehabilitación a domicilio puede ayudar a que los pacientes que estuvieron infectados puedan recuperarse más rápido después que sale de la hospitalización.

Antes de ser incluido en el estudio queremos que usted tome su tiempo de leer este Consentimiento Informado con detenimiento. En este documento es probable que pueda encontrar palabras que no sean de fácil compresión, por lo que le pedimos que haga las preguntas que considere necesarias para entender claramente en qué consistirá la participación en el estudio. En caso de que usted ACEPTE participar le daremos una copia de este documento cuando este cuente con todas las firmas.

PROPÓSITO DEL ESTUDIO

Usted ha sido seleccionado para participar en este estudio debido a que ha estado hospitalizado y ha requerido oxígeno para superar la enfermedad COVID-19. Varios reportes de comunidades científicas alrededor del mundo indican que posterior al COVID-19 las personas podrían quedar con daños pulmonares y mentales, especialmente aquellas que requirieron hospitalización., Los programas de rehabilitación integral han demostrado ser efectivos en mejorar la distintos indicadores de salud en pacientes con distintas enfermedades respiratorias, por lo que podrían también ayudar a disminuir las consecuencias que pueda causar por el COVID-19 en la salud a largo plazo.

Este estudio contará con 108 participantes que serán divididos en 2 grupos al azar. A un grupo (control) de participantes se les realizará evaluaciones periódicas para determinar el estado de sus pulmones y su salud mental en un periodo de 3 meses desde que sale del hospital. Al otro grupo (intervención) de participantes, además de las evaluaciones periódicas, se le brindará un programa de rehabilitación respiratoria y consejería psicológica en su domicilio de 6 semanas de duración. Usted participará en el estudio solamente si es su voluntad y tiene la posibilidad abierta a retirarse en cualquier momento, sin que esto cause problemas a su atención.

PROCEDIMIENTOS
Antes de ser incluido definitivamente en el estudio debe pasar por una evaluación de preselección que nos permita evaluar si cumple las características para ingresar al estudio. En esta fase de preselección se le hará una entrevista para colectar datos generales y antecedentes de enfermedades que haya tenido. También se realizarán las pruebas de calidad de vida, salud mental, función de los pulmones y resistencia física. Mediante estas evaluaciones usted podrá ser considerado apto para el estudio y recibirá la invitación para ser parte de este, así como el consentimiento informado general donde se especifican los procedimientos a realizarse en caso usted participe del estudio.

La prueba de estado cognitivo se realizará mediante una encuesta que evalúa su estado cognitivo actual, esta encuesta será realizada por un entrevistador a cargo y tendrá una duración aproximada de 10 minutos.

La prueba de caminata de 6 minutos será realizada en el hospital con la monitorización de una enfermera y un médico. Durante su realización se recolectarán sus funciones vitales al inicio, durante y al finalizar la prueba, así como la distancia total recorrida y se registrará además cualquier otro evento que pueda haber ocurrido. Esta prueba puede tomar en torno a 30 minutos para ser realizada.

La evaluación de indicadores de salud mental se realizará mediante la aplicación de tres cuestionarios que evalúan síntomas de depresión, ansiedad y estrés causados por eventos traumatizantes como la hospitalización. Estos cuestionarios serán administrados por un entrevistador. La aplicación de los tres cuestionarios tendráá una duración aproximada de 20 minutos.

La evaluación de indicadores de calidad de vida se realizará mediante la aplicación de dos cuestionarios. Estos cuestionarios serán administrados por un entrevistador y tendrán una duración de 15 minutos.

La espirometría es un procedimiento indoloro que la función de los pulmones. Evalúa el flujo de aire al medir la cantidad de aire que usted exhala y qué tan rápidamente lo hace. Mientras usted esté sentado, respirará dentro de una boquilla que va conectada a un instrumento llamado espirómetro, que se encarga de registrar la cantidad y frecuencia del aire inspirado y espirado durante un cierto período de tiempo. Este procedimiento se realizará en el hospital por una enfermera que registrará los datos recolectados y tendrá una duración de 30 minutos.

La presión arterial se medirá mientras usted esté sentado. Primero se medirá la presión arterial en ambos brazos. En el brazo que se obtenga la lectura más alta, se repetirá la medición dos veces más. Este procedimiento puede causar una sensación de presión leve en el brazo que dura aproximadamente 20 segundos y no causa dolor. La toma de la presión arterial tendrá una duración de 15 minutos.

RIESGOS Y/O INCOMODIDADES
Los riesgos del estudio son leves, poco frecuentes y raramente visibles, sin embargo, no excluimos la posibilidad de que se presenten efectos graves debido a que aún se desconoce cuáles son los daños que puede dejar el COVID-19 en su organismo a largo plazo y que podrían causar problemas respiratorios y/o cardiacos Sin embargo, los procedimientos a utilizarse en este estudio para evaluar periódicamente los indicadores de salud han demostrado ser altamente seguros, y en caso de presentar efectos adversos estos suelen ser relativamente leves y no representan un daño significativo para su salud. Además de que se

tomarán múltiples precauciones y medidas preventivas necesarias para evitar causar algún eventual daño a su integridad física.

En relación directa a los procedimientos a realizar para la evaluación de los indicadores requeridos para la preselección se podrían presentar riesgos y/o incomodidades tales como:

La caminata de 6 minutos que es un procedimiento seguro incluso en pacientes que han tenido ataques cardiacos recientes. Los síntomas más frecuentes incluyen sensación de falta de aire intolerable, calambres musculares, sudoración, vértigo, palidez, sensación de desvanecimiento, descenso del nivel de oxígeno y dolor torácico. Si el paciente presenta cualquiera de estos síntomas la prueba se detendrá inmediatamente. Complicaciones que no han sido reportadas, pero cabe la posibilidad de que se presenten, incluyen complicaciones cardiovasculares como desmayos, problemas del corazón y parada cardiorrespiratoria que podría llevar a daños mayores hasta la muerte. No obstante, las posibilidades de que se presenten problemas tales como los mencionados anteriormente son casi nulas. Además, como precaución al momento de la evaluación estará disponible un médico con preparación en primeros auxilios, un equipo de reanimación cardiopulmonar un balón de oxígeno que serán utilizados según necesidad.

En relación a la realización de la prueba de función de los pulmones (espirometría)
La prueba que mide el flujo del aire (espirometría) es un proceso generalmente seguro, no obstante, hay posibilidad de que experimente mareos o en casos muy severos hasta desmayos debido al esfuerzo respiratorio requerido. Ocasionalmente puede existir fuga de orina. Para evitar estas complicaciones, el procedimiento se realizará con los pacientes sentados en una silla estacionaria para minimizar los riesgos.

En relación a la aplicación de los cuestionarios
No existen riesgos asociados a la aplicación de los cuestionarios.

BENEFICIOS
Todas las pruebas realizadas en este estudio para evaluar su salud no le involucrarán gasto alguno y los resultados de las evaluaciones se le brindarán gratuitamente. Así mismo, si se detectara alguna alteración moderada-severa de salud mental será referirá a un psiquiatra para que le brinde el tratamiento oportuno.

COMPENSACIÓN
La participación es totalmente voluntaria, no se le dará dinero por participar en el proceso de preselección de este estudio ni en el estudio en sí.

CONFIDENCIALIDAD
Se registrarán todas sus respuestas a las preguntas y resultados de la evaluación según los formularios que se le proporcionen. Cada documento conde registrar información sobre usted será debidamente codificado alfanuméricamente para guardar el anonimato. Su nombre y datos personales serán de acceso y conocimiento exclusivo de la persona que lo examina y/o los investigadores que realizan el estudio. Sus registros de estudio (historia clínica, resultados de exámenes) no serán puestos a disposición de cualquier persona extraña al estudio sin su consentimiento. Sin embargo, esta información si podrá revisada por el personal que monitoriza el protocolo, así como por las instituciones que supervisan y regulan los trabajos de investigación para garantizar los aspectos éticos para su seguridad y el buen funcionamiento del estudio.

PREGUNTAS

Si presenta alguna duda y/o pregunta acerca de este estudio, puede ponerse en contacto con los profesionales encargados de su cuidado y/o con el investigador Anderson Nelver Soriano Moreno a su número de celular 999113385 o mediante comunicación escrita al e-mail anderson.soriano@upch.pe. Si tiene preguntas sobre los aspectos éticos del estudio, o cree que ha sido tratado injustamente puede contactar al Dr. Jorge Luis Hung Yep, Presidente del Comité Institucional de Ética en Investigación del Hospital Cayetano Heredia, a través de su número de celular 995732330, o con la Dra. Frine Samalvides Cuba, presidenta del Comité Institucional de Ética en Investigación de la Universidad Peruana Cayetano Heredia al teléfono 01-3190000, anexo 201355 o al correo electrónico: duict.cieh@oficinas-upch.pe

PARTICIPACION VOLUNTARIA

Su participación en este estudio es completamente voluntaria. Usted tiene la posibilidad de negarse a participar o puede dejar de participar en cualquier momento o etapa de este. Si deja de participar se le continuará dando el tratamiento estándar seguido por el Hospital y no habrá ningún tipo de afectación en su atención médica y la calidad de esta en el futuro o en su participación en futuros estudios.

CONSENTIMIENTO

He leído detenidamente este documento de consentimiento informado y he tenido la oportunidad de conversar, despejar mis dudas y hacer preguntas acerca del estudio en el que deseo participar, doy mi autorización expresa y totalmente voluntaria para la evaluación del proceso de preselección. Sé que voy a recibir una copia de este documento de consentimiento una vez que haya sido firmado.

Si Acepto ( )   No Acepto ( )

Autorizo a ser re-contactado para futuras investigaciones relacionadas con este estudio, previa evaluación de proyecto de investigación por el Comité de ética. En caso marque sí, deje su número de teléfono y dirección al final del documento.

Sí Acepto ( )   No Acepto ( )

Firmas:

Huella Digital

___________________________	________________________	________________	______________
Nombre del Paciente	Firma	Fecha	Hora

(en vez de firma si el participante no sabe leer ni escribir,
Podrá poner la huella digital de su dedo índice derecho)

Dirección: ______________________________Distrito:_________Dep/Provincia:________
Teléfono: _________________

___________________________	________________________	________________	______________
Nombre del Testigo	Firma	Fecha	Hora

(requerido solo si el participante no sabe leer ni escribir)

Nombre del Entrevistador	Firma	Fecha	Hora

CONSENTIMIENTO INFORMADO PARA PARTICIPAR EN UN ESTUDIO DE INVESTIGACIÓN

INSTITUCIONES: **Universidad Peruana Cayetano Heredia (Lima, Perú)**

Johns Hopkins University (Baltimore, Maryland US)

The London School of Hygiene & Tropical Medicine

INVESTIGADOR: **Stella Maria Hartinger Peña, PhD**
E-MAIL: **stella.hartinger.p@upch.pe**

ESTUDIO: **Prevalencia de alteración de la función pulmonar post infección por COVID-19 e impacto de la participación en un programa piloto de rehabilitación integral a corto y mediano plazo.**

Lo invitamos a participar en un estudio de investigación que busca evaluar cuál es el daño que el COVID-19 puede dejar en los pulmones y en la salud mental. También busca ver si un programa de rehabilitación a domicilio puede ayudar a que los pacientes que estuvieron infectados puedan recuperarse más rápido después que sale de la hospitalización.

Antes de ser incluido en el estudio queremos que usted tome su tiempo de leer este Consentimiento Informado con detenimiento. En este documento es probable que pueda encontrar palabras que no sean de fácil compresión, por lo que le pedimos que haga las preguntas que considere necesarias para entender claramente en qué consistirá la participación en el estudio. En caso de que usted ACEPTE participar le daremos una copia de este documento cuando este cuente con todas las firmas.

PROPÓSITO DEL ESTUDIO

Usted ha sido seleccionado para participar en este estudio debido a que ha estado hospitalizado y ha requerido oxígeno para superar la enfermedad COVID-19. Varios reportes de comunidades científicas alrededor del mundo indican que posterior al COVID-19 las personas podrían quedar con daños pulmonares y mentales, especialmente aquellas que requirieron hospitalización. Los programas de rehabilitación han demostrado ser efectivos en mejorar distintos otros indicadores de salud en pacientes con distintas enfermedades respiratorias, por lo que podrían también ayudar a disminuir las consecuencias que pueda causar el COVID-19 en la salud a largo plazo.

PROCEDIMIENTOS

Después de haber pasado la fase de preselección y se haya considerado apto por cumplir los criterios establecidos por el estudio, se completará el proceso de selección y será incluido oficialmente como participante mediante la firma de este Consentimiento Informado. Posteriormente, será asignado al azar a uno de los dos grupos del estudio. A un grupo de participantes (control) se les realizará evaluaciones periódicas para evaluar la salud de sus pulmones y su salud mental en un periodo de 3 meses desde que salen del hospital. Al otro grupo (intervención) de participantes, además de las evaluaciones periódicas, se les brindará un programa de rehabilitación respiratoria y consejería psicológica en su domicilio de 6 semanas de duración.

En relación directa a la intervención-

La rehabilitación respiratoria consistirá en 12 sesiones de ejercicios respiratorios de baja intensidad que realizará en su hogar con ayuda de un fisioterapeuta. Estas sesiones serán brindadas en una frecuencia de 2 veces por semana y tendrán una duración aproximada de 40 minutos por sesión. El horario en que se brindarán las sesiones de rehabilitación dependerá de su disponibilidad. En la primera sesión de rehabilitación se le entregará los insumos necesarios para la realización de los ejercicios (pesa de 1kg, dispositivo manual de presión positiva). La rehabilitación consistirá en ejercicios que seguirán un protocolo previamente establecido que han demostrado eficacia en estudios previos con pacientes adultos mayores sobrevivientes al COVID-19. Estos ejercicios involucran el entrenamiento de los músculos que le ayudan a respirar, a mejorar la tos y estiramientos de los brazos. Los ejercicios se realizarán a su propio ritmo con ayuda del profesional capacitado. No tendrá que hacer sobresfuerzo que pueda repercutir negativamente en su salud.

El soporte psicológico consistirá en 6 sesiones de terapia hablada brindadas por psicólogos mediante llamada telefónica o videollamada. Cada sesión tendrá una duración de 1 hora y se realizarán con una frecuencia de 1 vez por semana. El horario en que se brindarán las sesiones se coordinará según su disponibilidad. El objetivo de estas terapias es ayudarlo en adquirir habilidades para la resolución efectiva de problemas y para hacer frente a los estresores de la vida a fin de atenuar los efectos negativos que pueda haber tenido el COVID-19 a nivel mental.

Las visitas de seguimiento se realizarán en el Hospital Nacional Cayetano Heredia. Durante los 3 meses de seguimiento, usted tendrá que acudir en 2 ocasiones al hospital para realizar la evaluación del estado de sus pulmones y su corazón mediante distintas pruebas que se detallan a continuación. Cada visita tendrá una duración aproximada de 4 horas. La primera visita de seguimiento se realizará al cabo de 6 semanas desde que fue incluido en el estudio donde se le hizo una primera evaluación. La segunda visita de seguimiento será la última visita y será 6 semanas después de su última evaluación (es decir a la semana 12 de haber iniciado el estudio).

En cada visita se realizará una prueba que mide el flujo del aire de los pulmones, llamada **espirometría**, una prueba de caminata de 6 minutos, y se aplicarán otros cuestionarios para evaluar la salud respiratoria, calidad de vida y estado cognitivo. Los procedimientos se detallan a continuación.

En relación a las pruebas para la evaluación de indicadores-

La prueba de caminata de 6 minutos, es una prueba que permite evaluar el estado de su corazón y pulmones. Usted tendrá que caminar durante 6 minutos seguidos para medir cuanta distancia puede caminar. Esta prueba será realizada en el hospital con la monitorización de un médico y enfermera. Durante su realización se recolectarán sus funciones vitales al inicio, durante y al finalizar la prueba, así como la distancia total recorrida y cualquier observación.

La espirometría es un procedimiento indoloro que evalúa el flujo de aire al medir la cantidad de aire que usted exhala y qué tan rápidamente lo hace. Mientras usted esté sentado, respirará dentro de una boquilla que va conectada a un instrumento llamado espirómetro, que se encarga de registrar la cantidad y frecuencia del aire inspirado y espirado durante un cierto período de tiempo. Este procedimiento se realizará en el hospital por una enfermera que registrarán los datos recolectados.

La evaluación de indicadores de salud mental se realizará cada 2 semanas durante las primeras 12 semanas de seguimiento mediante la aplicación de tres cuestionarios que evalúan la presencia de síntomas de depresión, ansiedad y estrés. La toma de estos cuestionarios durará aproximadamente 30 minutos y serán administrados por psicólogos mediante vía telefónica quienes registrarán las respuestas obtenidas.

La evaluación de los síntomas respiratorios se realizará también mediante un cuestionario validado para el propósito y será aplicado durante su visita de seguimiento en el hospital por un entrevistador quien registrará las respuestas obtenidas. La aplicación del cuestionario tomará un promedio de 20 minutos.

La evaluación de indicadores de calidad de vida se realizará mediante dos cuestionarios validados para medir la calidad de vida. Estos cuestionarios serán administrados durante su visita de seguimiento en el hospital por un entrevistador que registrará las respuestas obtenidas. La aplicación del cuestionario tomará un promedio de 20 minutos.

La presión arterial se medirá mientras usted esté sentado. Primero se medirá la presión arterial en ambos brazos. En el brazo que se obtenga la lectura más alta, se repetirá la medición dos veces más. Este procedimiento puede causar una sensación de presión leve en el brazo que dura aproximadamente 20 segundos y no causa dolor. La toma de la presión arterial tendrá una duración de 15 minutos.

RIESGOS Y/O INCOMODIDADES
Los riesgos del estudio son leves, poco frecuentes y raramente visibles, sin embargo, no excluimos la posibilidad de que se presenten efectos graves debido a que aún se desconoce cuáles son los daños que puede dejar el COVID-19 en su organismo y que podrían causar problemas respiratorios y/o cardiacos .Sin embargo, los procedimientos a utilizarse en este estudio para evaluar periódicamente los indicadores de salud han demostrado ser altamente seguros, y en caso de presentar efectos adversos, estos suelen ser relativamente leves y no representan un daño significativo para su salud. Además de que se tomarán múltiples precauciones y medidas preventivas necesarias para evitar causar algún eventual daño a su integridad física.

En relación directa a los procedimientos a realizar para la evaluación de los indicadores requeridos por el estudio se podrían presentar riesgos y/o incomodidades tales como:

La caminata de 6 minutos que es un procedimiento seguro incluso en pacientes que han tenido ataques cardiacos recientes. Los síntomas más frecuentes incluyen sensación de falta de aire intolerable, calambres musculares, sudoración, vértigo, palidez, sensación de desvanecimiento, descenso del nivel de oxígeno y dolor torácico. Si el paciente presentara cualquiera de estos síntomas la prueba se detendrá inmediatamente. Complicaciones que no han sido reportadas, pero cabe la posibilidad de que se presenten, incluyen complicaciones cardiovasculares como desmayos, problemas del corazón y parada cardiorrespiratoria que podría llevar a daños mayores hasta la muerte. No obstante, las posibilidades de que se presenten problemas tales como los mencionados anteriormente son casi nulas. Además, como precaución por esta razón al momento de la evaluación estará disponible un médico con preparación en primeros auxilios, un equipo de reanimación cardiopulmonar un balón de oxígeno que serán utilizados según necesidad.

La prueba que mide el flujo del aire (espirometría) es un proceso generalmente seguro, no obstante, hay posibilidad de que experimente mareos o en casos muy severos hasta desmayos debido al esfuerzo respiratorio requerido. Ocasionalmente puede existir fuga de orina. Para evitar estas complicaciones, el procedimiento se realizará con los pacientes sentados en una silla estacionaria para minimizar los riesgos. Para evaluar los eventos adversos inesperados que puedan estar asociados al protocolo de estudio se vigilará a los participantes de forma cercana. Los investigadores del estudio determinarán si los eventos adversos clínicos que ocurran durante la duración del estudio son debidos a los procedimientos o la intervención. Existe un riesgo de angustia psicológica al tener el conocimiento de que pueda tener una afectación pulmonar como consecuencia del COVID-19.

En relación al programa de rehabilitación

La rehabilitación respiratoria temprana ha demostrado ser altamente segura, eficaz e importante en la recuperación de los pacientes. Sin embargo, en raras ocasiones la actividad física puede causar problemas como lesiones en los músculos y los huesos. Así mismo, en caso de pacientes que han tenido daño en los pulmones, como los pacientes COVID-19, pueden presentar disminución del oxígeno. Por esta razón, previo inicio de la terapia, se tomarán las funciones vitales, para evaluar si el participante se encuentra en condición de realizar los ejercicios. Así mismo, durante toda la terapia, llevará puesto un equipo posicionado en el dedo índice que nos permitirá monitorizar el oxígeno y la frecuencia de pulso. Si se detectara que el oxígeno disminuye en la sangre o la frecuencia de pulso se eleva demasiado, el equipo de rehabilitación pulmonar detendrá la sesión de inmediato para brindarle la asistencia correspondiente. También se contará con un balón de oxígeno para ser suministrado inmediatamente en caso de que se requiera.

En relación a la aplicación de los cuestionarios

No existen riesgos asociados a la aplicación de los cuestionarios de seguimiento.

BENEFICIOS

Todas las pruebas realizadas en este estudio para evaluar su salud no le involucrarán gasto alguno y los resultados de las evaluaciones se le brindarán gratuitamente. Así mismo, si se detectara alguna alteración en salud se le informará y recomendará acudir al profesional correspondiente para que le brinde el tratamiento oportuno. La terapia de rehabilitación respiratoria y psicológica tampoco le involucrará gasto alguno.

Los datos derivados podrían ser de gran beneficio para un posible diagnóstico médico oportuno de alguna otra enfermedad no relacionada a la intervención o autoconocimiento de sus condiciones de salud. Un seguimiento cercano de sus signos vitales como frecuencia cardiaca, presión arterial, capacidad pulmonar y demás por parte de un médico es de suma importancia para poder derivar recomendaciones específicas para el cuidado de su salud. La evaluación psicológica periódica también podrá ayudar a reconocer áreas de enfoque específico en el soporte gratuito que se le brindará.

COMPENSACIÓN

La participación es totalmente voluntaria, no se le dará dinero por participar en este estudio. El estudio se encargará de cubrir los costos relacionados con las pruebas que se realizarán para la evaluación de la salud de los pulmones (espirometría, prueba de caminata de 6 minutos) y el programa de rehabilitación . Cualquier daño que se determine que haya sido causado por procedimientos del estudio será cubierto. Los gastos de movilización al hospital para las visitas de seguimiento serán cubiertos por el estudio si fuera necesario. No se

esperan daños provocados por este estudio de investigación, sin embargo, en caso llegará a suceder este será cubierto por una póliza de seguro.

ALTERNATIVAS

Actualmente no hay un modelo o protocolo de rehabilitación establecido por alguna autoridad sanitaria en el Perú para los pacientes COVID-19, más que las recomendaciones de autocuidado de la salud mental en las personas afectadas y campañas de información en la población. Si usted no desea participar en el estudio seguirá con la atención brindada por la entidad responsable de su salud.

CONFIDENCIALIDAD

Se registrarán todos los datos que provengan de las respuestas de los cuestionarios y evaluaciones que se le realizarán. Cada documento encargado de registrar información sobre usted será debidamente codificado alfanuméricamente para guardar el anonimato. Su nombre y datos personales serán de acceso y conocimiento exclusivo de la persona que lo examina y/o los investigadores que realizan el estudio. Sus registros de estudio (historia clínica, resultados de exámenes) no serán puestos a disposición de cualquier persona extraña al estudio sin su consentimiento. Sin embargo, esta información si podrá revisada por el personal que monitoriza el protocolo, así como por las instituciones que supervisan y regulan los trabajos de investigación para garantizar los aspectos éticos para su seguridad y el buen funcionamiento del estudio.

PREGUNTAS

Si presenta alguna duda y/o pregunta acerca de este estudio, puede ponerse en contacto con los profesionales encargados de su cuidado y/o con el investigador Anderson Nelver Soriano Moreno a su número de celular 999113385 o mediante comunicación escrita al e-mail anderson.soriano@upch.pe. Si tiene preguntas sobre los aspectos éticos del estudio, o cree que ha sido tratado injustamente puede contactar al Dr. Jorge Luis Hung Yep, Presidente del Comité Institucional de Ética en Investigación del Hospital Cayetano Heredia, a través de su número de celular 995732330, o con la Dra. Frine Samalvides Cuba, presidenta del Comité Institucional de Ética en Investigación de la Universidad Peruana Cayetano Heredia al teléfono 01-3190000, anexo 201355 o al correo electrónico: duict.cieh@oficinas-upch.pe

PARTICIPACION VOLUNTARIA

Su participación en este estudio es completamente voluntaria. Usted tiene la posibilidad de negarse a participar o puede dejar de participar en cualquier momento o etapa de este. Si deja de participar se le continuará dando el tratamiento estándar seguido por el Hospital y esto no afectará de ninguna forma la calidad de la atención que se le viene dando ni su participación en futuros estudios.

CONDICIONES PARA EL RETIRO

Se procederá al retiro del participante en las siguientes circunstancias específicamente:

En la prueba de caminata de 6 min.- Si presentara alguna complicación cardiovascular durante la realización de la prueba de caminata de 6 minutos ya sea en la medición realizada al inicio en la fase de preselección o en alguna de las evaluaciones de seguimiento.

En la rehabilitación respiratoria.- Si durante la evaluación de las funciones vitales, previo inicio de los ejercicios respiratorios, presentara un oxígeno muy bajo o frecuencia de pulso muy elevada la terapia se reprogramará para otro día. Complicaciones cardiovasculares durante la realización de los ejercicios respiratorios involucrarán una exclusión definitiva del estudio

En el soporte psicológico.- Intención suicida o psicosis detectada durante alguna de las evaluaciones psicológicas involucrará una exclusión definitiva.

CONSENTIMIENTO

He leído detenidamente este documento de consentimiento informado y he tenido la oportunidad de conversar, despejar mis dudas y hacer preguntas acerca del estudio en el que acepto participar. Doy mi autorización expresa y totalmente voluntaria para participar en este estudio. Sé que voy a recibir una copia de este documento de consentimiento una vez que haya sido firmado.

Sí Acepto ( )   No Acepto ( )

Autorizo a ser re-contactado para futuras investigaciones relacionadas con este estudio, previa evaluación de proyecto de investigación por el Comité de ética. En caso marque sí, deje su número de teléfono y dirección al final del documento.

Sí Acepto ( )   No Acepto ( )

Firmas:

Huella Digital

________________________ ____________________ _____________ ___________

Nombre del Paciente   Firma   Fecha   Hora

(en vez de firma si el participante no sabe leer ni escribir,
Podrá poner la huella digital de su dedo índice derecho)

Dirección: ______________________________Distrito:_________Dep/Provincia:________
Teléfono: _________________

________________________ ____________________ _____________ ___________

Nombre del Testigo   Firma   Fecha   Hora

(requerido solo si el participante no sabe leer ni escribir)

________________________ ____________________ _____________ ___________

Nombre del Entrevistador   Firma   Fecha   Hora
