# Supplementary material for: Home-Based Respiratory Physiotherapy and Telephone-Based Psychological Support for COVID-19 Survivors in Peru: Protocol for a Randomized Controlled Trial
Source: JMIR Res Protoc. 2022 Oct 24;11(10):e36001. doi: 10.2196/36001 (PMC9595592; doi:10.2196/36001)
Supplement: Multimedia Appendix 2 [file resprot_v11i10e36001_app2.pdf]

CONSORT-EHEALTH (V 1.6.1) - Submission/Publication Form

The CONSORT-EHEALTH checklist is intended for authors of randomized trials evaluating web-based and Internet-based applications/interventions, including mobile interventions, electronic games (incl multiplayer games), social media, certain telehealth applications, and other interactive and/or networked electronic applications. Some of the items (e.g. all subitems under item 5 - description of the intervention) may also be applicable for other study designs.

The goal of the CONSORT EHEALTH checklist and guideline is to be
a) a guide for reporting for authors of RCTs,
b) to form a basis for appraisal of an ehealth trial (in terms of validity)

CONSORT-EHEALTH items/subitems are MANDATORY reporting items for studies published in the Journal of Medical Internet Research and other journals / scientific societies endorsing the checklist.

Items numbered 1., 2., 3., 4a., 4b etc are original CONSORT or CONSORT-NPT (non-pharmacologic treatment) items.
Items with Roman numerals (i., ii, iii, iv etc.) are CONSORT-EHEALTH extensions/clarifications.

As the CONSORT-EHEALTH checklist is still considered in a formative stage, we would ask that you also RATE ON A SCALE OF 1-5 how important/useful you feel each item is FOR THE PURPOSE OF THE CHECKLIST and reporting guideline (optional).

Mandatory reporting items are marked with a red *.
In the textboxes, either copy & paste the relevant sections from your manuscript into this form - please include any quotes from your manuscript in QUOTATION MARKS,
or answer directly by providing additional information not in the manuscript, or elaborating on why the item was not relevant for this study.

YOUR ANSWERS WILL BE PUBLISHED AS A SUPPLEMENTARY FILE TO YOUR PUBLICATION IN JMIR AND ARE CONSIDERED PART OF YOUR PUBLICATION (IF ACCEPTED).
Please fill in these questions diligently. Information will not be copyedited, so please use proper spelling and grammar, use correct capitalization, and avoid abbreviations.

DO NOT FORGET TO SAVE AS PDF _AND_ CLICK THE SUBMIT BUTTON SO YOUR ANSWERS ARE IN OUR DATABASE !!!

Citation Suggestion (if you append the pdf as Appendix we suggest to cite this paper in the caption):
Eysenbach G, CONSORT-EHEALTH Group
[illegible]
J Med Internet Res 2011;13(4):e126

Estás editando tu respuesta. Si compartes esta URL, otros usuarios también podrán editar la respuesta.
RELLENAR UNA RESPUESTA NUEVA

URL: http://www.jmir.org/2011/4/e126/
doi: 10.2196/jmir.1923
PMID: 22209829

ahcl1234@gmail.com (no compartidos) Cambiar de cuenta

Volver a enviar para guardar

*Obligatorio

Your name *

First Last

Abdiel H. Coico-Lama

Primary Affiliation (short), City, Country *

University of Toronto, Toronto, Canada

Epidemiological and Clinical Research Unit, Sc

Your e-mail address *

abc@gmail.com

abdielcoico@upeu.edu.pe

Title of your manuscript *

Provide the (draft) title of your manuscript.

Effects of home-based intervention of respiratory physiotherapy and mobile-based psychological support for COVID-19 survivors: Protocol for a randomized controlled trial

Estás editando tu respuesta. Si compartes esta URL, otros usuarios también podrán editar la respuesta.

RELLENAR UNA RESPUESTA NUEVA

Name of your App/Software/Intervention *

If there is a short and a long/alternate name, write the short name first and add the long name in brackets.

WAYRA

Evaluated Version (if any)

e.g. "V1", "Release 2017-03-01", "Version 2.0.27913"

Tu respuesta

Language(s) *

What language is the intervention/app in? If multiple languages are available, separate by comma (e.g. "English, French")

Spanish

URL of your Intervention Website or App

e.g. a direct link to the mobile app on app in appstore (itunes, Google Play), or URL of the website. If the intervention is a DVD or hardware, you can also link to an Amazon page.

Tu respuesta

URL of an image/screenshot (optional)

Tu respuesta

Estás editando tu respuesta. Si compartes esta URL, otros usuarios también podrán editar la respuesta.

RELLENAR UNA RESPUESTA NUEVA

Accessibility *

Can an enduser access the intervention presently?

- ○ access is free and open
- ○ access only for special usergroups, not open
- ○ access is open to everyone, but requires payment/subscription/in-app purchases
- ◉ app/intervention no longer accessible
- ○ Otro:

Primary Medical Indication/Disease/Condition *

e.g. "Stress", "Diabetes", or define the target group in brackets after the condition, e.g. "Autism (Parents of children with)", "Alzheimers (Informal Caregivers of)"

Confirmed cases of COVID-19, diagnosed by s[illegible]

Primary Outcomes measured in trial *

comma-separated list of primary outcomes reported in the trial

The change in six-minute walk distance (6MW[illegible]

Secondary/other outcomes

Are there any other outcomes the intervention is expected to affect?

Lung function (FEV1, FVC, FEV1/FVC), mental health (PHQ-9, GAD-7, and IES-R), quality of life (SF-36 v2, and EQ-5D), respiratory symptoms (SGRQ).

Estás editando tu respuesta. Si compartes esta URL, otros usuarios también podrán editar la respuesta.

RELLENAR UNA RESPUESTA NUEVA

Recommended "Dose" *

What do the instructions for users say on how often the app should be used?

- ( ) Approximately Daily
- (x) Approximately Weekly
- ( ) Approximately Monthly
- ( ) Approximately Yearly
- ( ) "as needed"
- ( ) Otro:

Approx. Percentage of Users (starters) still using the app as recommended after 3 months *

- (x) unknown / not evaluated
- ( ) 0-10%
- ( ) 11-20%
- ( ) 21-30%
- ( ) 31-40%
- ( ) 41-50%
- ( ) 51-60%
- ( ) 61-70%
- ( ) 71%-80%
- ( ) 81-90%
- ( ) 91-100%
- ( ) Otro:

Estás editando tu respuesta. Si compartes esta URL, otros usuarios también podrán editar la respuesta.

RELLENAR UNA RESPUESTA NUEVA

Overall, was the app/intervention effective? *

- ◯ yes: all primary outcomes were significantly better in intervention group vs control
- ◉ partly: SOME primary outcomes were significantly better in intervention group vs control
- ◯ no statistically significant difference between control and intervention
- ◯ potentially harmful: control was significantly better than intervention in one or more outcomes
- ◯ inconclusive: more research is needed
- ◯ Otro:

Article Preparation Status/Stage *

At which stage in your article preparation are you currently (at the time you fill in this form)

- ◯ not submitted yet - in early draft status
- ◯ not submitted yet - in late draft status, just before submission
- ◉ submitted to a journal but not reviewed yet
- ◯ submitted to a journal and after receiving initial reviewer comments
- ◯ submitted to a journal and accepted, but not published yet
- ◯ published
- ◯ Otro:

Estás editando tu respuesta. Si compartes esta URL, otros usuarios también podrán editar la respuesta.

RELLENAR UNA RESPUESTA NUEVA

Journal *

If you already know where you will submit this paper (or if it is already submitted), please provide the journal name (if it is not JMIR, provide the journal name under "other")

- ○ not submitted yet / unclear where I will submit this
- ○ Journal of Medical Internet Research (JMIR)
- ○ JMIR mHealth and UHealth
- ○ JMIR Serious Games
- ○ JMIR Mental Health
- ○ JMIR Public Health
- ○ JMIR Formative Research
- ◉ Other JMIR sister journal
- ○ Otro:

Is this a full powered effectiveness trial or a pilot/feasibility trial? *

- ◉ Pilot/feasibility
- ○ Fully powered

Manuscript tracking number *

If this is a JMIR submission, please provide the manuscript tracking number under "other" (The ms tracking number can be found in the submission acknowledgement email, or when you login as author in JMIR. If the paper is already published in JMIR, then the ms tracking number is the four-digit number at the end of the DOI, to be found at the bottom of each published article in JMIR)

- ○ no ms number (yet) / not (yet) submitted to / published in JMIR
- ◉ Otro: 36001

Estás editando tu respuesta. Si compartes esta URL, otros usuarios también podrán editar la respuesta. RELLENAR UNA RESPUESTA NUEVA

TITLE AND ABSTRACT

1a) TITLE: Identification as a randomized trial in the title

1a) Does your paper address CONSORT item 1a? *

I.e does the title contain the phrase "Randomized Controlled Trial"? (if not, explain the reason under "other")

- (•) yes
- ( ) Otro:

1a-i) Identify the mode of delivery in the title

Identify the mode of delivery. Preferably use “web-based” and/or “mobile” and/or “electronic game” in the title. Avoid ambiguous terms like “online”, “virtual”, “interactive”. Use “Internet-based” only if Intervention includes non-web-based Internet components (e.g. email), use “computer-based” or “electronic” only if offline products are used. Use “virtual” only in the context of “virtual reality” (3-D worlds). Use “online” only in the context of “online support groups”. Complement or substitute product names with broader terms for the class of products (such as “mobile” or “smart phone” instead of “iphone”), especially if the application runs on different platforms.

	1	2	3	4	5	
subitem not at all important	( )	( )	( )	( )	(•)	essential

Borrar selección

Does your paper address subitem 1a-i? *

Copy and paste relevant sections from manuscript title (include quotes in quotation marks "like this" to indicate direct quotes from your manuscript), or elaborate on this item by providing additional information not in the ms, or briefly explain why the item is not applicable/relevant for your study

Estás editando tu respuesta. Si compartes esta URL, otros usuarios también podrán editar la respuesta. RELLENAR UNA RESPUESTA NUEVA

1a-ii) Non-web-based components or important co-interventions in title

Mention non-web-based components or important co-interventions in title, if any (e.g., “with telephone support”).

	1	2	3	4	5	
subitem not at all important	○	○	○	○	◉	essential

Borrar selección

Does your paper address subitem 1a-ii?

Copy and paste relevant sections from manuscript title (include quotes in quotation marks "like this" to indicate direct quotes from your manuscript), or elaborate on this item by providing additional information not in the ms, or briefly explain why the item is not applicable/relevant for your study

"telephone-based psychological support"

1a-iii) Primary condition or target group in the title

Mention primary condition or target group in the title, if any (e.g., “for children with Type I Diabetes”) Example: A Web-based and Mobile Intervention with Telephone Support for Children with Type I Diabetes: Randomized Controlled Trial

	1	2	3	4	5	
subitem not at all important	○	○	○	○	◉	essential

Borrar selección

Does your paper address subitem 1a-iii? *

Copy and paste relevant sections from manuscript title (include quotes in quotation marks "like this" to indicate direct quotes from your manuscript), or elaborate on this item by providing additional information not in the ms, or briefly explain why the item is not applicable/relevant for your study

Estás editando tu respuesta. Si compartes esta URL, otros usuarios también podrán editar la respuesta.

RELLENAR UNA RESPUESTA NUEVA

1b) ABSTRACT: Structured summary of trial design, methods, results, and conclusions

NPT extension: Description of experimental treatment, comparator, care providers, centers, and blinding status.

1b-i) Key features/functionalities/components of the intervention and comparator in the METHODS section of the ABSTRACT

Mention key features/functionalities/components of the intervention and comparator in the abstract. If possible, also mention theories and principles used for designing the site. Keep in mind the needs of systematic reviewers and indexers by including important synonyms. (Note: Only report in the abstract what the main paper is reporting. If this information is missing from the main body of text, consider adding it)

	1	2	3	4	5	
subitem not at all important	○	○	○	○	◉	essential

Borrar selección

Does your paper address subitem 1b-i? *

Copy and paste relevant sections from the manuscript abstract (include quotes in quotation marks "like this" to indicate direct quotes from your manuscript), or elaborate on this item by providing additional information not in the ms, or briefly explain why the item is not applicable/relevant for your study

"The intervention consisted of twelve at-home respiratory rehabilitation sessions and six telephone-based psychological sessions"

Estás editando tu respuesta. Si compartes esta URL, otros usuarios también podrán editar la respuesta.

RELLENAR UNA RESPUESTA NUEVA

1b-ii) Level of human involvement in the METHODS section of the ABSTRACT

Clarify the level of human involvement in the abstract, e.g., use phrases like “fully automated” vs. “therapist/nurse/care provider/physician-assisted” (mention number and expertise of providers involved, if any). (Note: Only report in the abstract what the main paper is reporting. If this information is missing from the main body of text, consider adding it)

	1	2	3	4	5	
subitem not at all important	○	○	○	○	◉	essential

Borrar selección

Does your paper address subitem 1b-ii?

Copy and paste relevant sections from the manuscript abstract (include quotes in quotation marks "like this" to indicate direct quotes from your manuscript), or elaborate on this item by providing additional information not in the ms, or briefly explain why the item is not applicable/relevant for your study

This part is not relevant to the study, because it is explained in the body of the manuscript.

1b-iii) Open vs. closed, web-based (self-assessment) vs. face-to-face assessments in the METHODS section of the ABSTRACT

Mention how participants were recruited (online vs. offline), e.g., from an open access website or from a clinic or a closed online user group (closed usergroup trial), and clarify if this was a purely web-based trial, or there were face-to-face components (as part of the intervention or for assessment). Clearly say if outcomes were self-assessed through questionnaires (as common in web-based trials). Note: In traditional offline trials, an open trial (open-label trial) is a type of clinical trial in which both the researchers and participants know which treatment is being administered. To avoid confusion, use “blinded” or “unblinded” to indicated the level of blinding instead of “open”, as “open” in web-based trials usually refers to “open access” (i.e. participants can self-enrol). (Note: Only report in the abstract what the main paper is reporting. If this information is missing from the main body of text, consider adding it)

	1	2	3	4	5	
subitem not at all important	○	○	○	○	◉	essential

Estás editando tu respuesta. Si compartes esta URL, otros usuarios también podrán editar la respuesta.

RELLENAR UNA RESPUESTA NUEVA

Does your paper address subitem 1b-iii?

Copy and paste relevant sections from the manuscript abstract (include quotes in quotation marks "like this" to indicate direct quotes from your manuscript), or elaborate on this item by providing additional information not in the ms, or briefly explain why the item is not applicable/relevant for your study

"open-label, unblinded, two-arm randomized controlled trial"

1b-iv) RESULTS section in abstract must contain use data

Report number of participants enrolled/assessed in each group, the use/uptake of the intervention (e.g., attrition/adherence metrics, use over time, number of logins etc.), in addition to primary/secondary outcomes. (Note: Only report in the abstract what the main paper is reporting. If this information is missing from the main body of text, consider adding it)

	1	2	3	4	5	
subitem not at all important	○	○	○	○	◉	essential

Borrar selección

Does your paper address subitem 1b-iv?

Copy and paste relevant sections from the manuscript abstract (include quotes in quotation marks "like this" to indicate direct quotes from your manuscript), or elaborate on this item by providing additional information not in the ms, or briefly explain why the item is not applicable/relevant for your study

"We recruited 108 participants aged 18–75 years who were discharged from the hospital after COVID-19 pneumonia that required >6 L/min of supplemental oxygen during treatment"

Estás editando tu respuesta. Si compartes esta URL, otros usuarios también podrán editar la respuesta.

RELLENAR UNA RESPUESTA NUEVA

1b-v) CONCLUSIONS/DISCUSSION in abstract for negative trials

Conclusions/Discussions in abstract for negative trials: Discuss the primary outcome - if the trial is negative (primary outcome not changed), and the intervention was not used, discuss whether negative results are attributable to lack of uptake and discuss reasons. (Note: Only report in the abstract what the main paper is reporting. If this information is missing from the main body of text, consider adding it)

	1	2	3	4	5	
subitem not at all important	○	○	○	○	◉	essential

Borrar selección

Does your paper address subitem 1b-v?

Copy and paste relevant sections from the manuscript abstract (include quotes in quotation marks "like this" to indicate direct quotes from your manuscript), or elaborate on this item by providing additional information not in the ms, or briefly explain why the item is not applicable/relevant for your study

This part is not relevant to the study in the abstract part, because it is explained in the body of the manuscript, specifically in the discussion section.

INTRODUCTION

2a) In INTRODUCTION: Scientific background and explanation of rationale

Estás editando tu respuesta. Si compartes esta URL, otros usuarios también podrán editar la respuesta. RELLENAR UNA RESPUESTA NUEVA

2a-i) Problem and the type of system/solution

Describe the problem and the type of system/solution that is object of the study: intended as stand-alone intervention vs. incorporated in broader health care program? Intended for a particular patient population? Goals of the intervention, e.g., being more cost-effective to other interventions, replace or complement other solutions? (Note: Details about the intervention are provided in “Methods” under 5)

	1	2	3	4	5	
subitem not at all important	○	○	○	○	◉	essential

Borrar selección

Does your paper address subitem 2a-i? *

Copy and paste relevant sections from the manuscript (include quotes in quotation marks "like this" to indicate direct quotes from your manuscript), or elaborate on this item by providing additional information not in the ms, or briefly explain why the item is not applicable/relevant for your study

"Considering that COVID-19 is a disease that results in short-, medium- and long-term physical and mental impairment and that limited rehabilitation strategies have been evaluated, this manuscript describes the design and methodology of a randomized controlled trial that aimed to evaluate the effects of integrated home-based respiratory physiotherapy combined with a telephone-based psychological intervention on pulmonary and mental health-related outcomes at 7 and 12 weeks after hospital discharge in patients with COVID-19 pneumonia. The primary objective of the trial was to compare 6-minute walk distances at 7 and 12 weeks after hospital discharge for COVID-19 pneumonia in participants of a combined 6-week home-based respiratory physiotherapy and telephone-based psychological rehabilitation program versus those who received usual care practices."

Estás editando tu respuesta. Si compartes esta URL, otros usuarios también podrán editar la respuesta.

RELLENAR UNA RESPUESTA NUEVA

2a-ii) Scientific background, rationale: What is known about the (type of) system

Scientific background, rationale: What is known about the (type of) system that is the object of the study (be sure to discuss the use of similar systems for other conditions/diagnoses, if appropiate), motivation for the study, i.e. what are the reasons for and what is the context for this specific study, from which stakeholder viewpoint is the study performed, potential impact of findings [2]. Briefly justify the choice of the comparator.

	1	2	3	4	5	
subitem not at all important	○	○	○	○	◉	essential

Borrar selección

Does your paper address subitem 2a-ii? *

Copy and paste relevant sections from the manuscript (include quotes in quotation marks "like this" to indicate direct quotes from your manuscript), or elaborate on this item by providing additional information not in the ms, or briefly explain why the item is not applicable/relevant for your study

"The COVID-19 pandemic is resulting in millions of survivors with long-term complications [1]. Complications are more frequent in patients who required hospitalization, and affect both physical and mental health [1]. Systematic reviews show that 24% to 36% and 40% to 58% of patients with COVID-19 report dyspnea and fatigue after acute illness, respectively [2,3]. The frequency of depression and anxiety in COVID-19 survivors ranges from 12% to 15% and 13% to 22%, respectively [2–5]. Although recommendations for post-COVID rehabilitation already exist [6–9], the evidence is scarce in different aspects. First, most studies have focused on pulmonary rehabilitation [10–12] but none evaluate a rehabilitation program that integrates the respiratory component with the psychological component. The frequencies of mental illness suggest that patients may benefit from physiological support. Second, no published clinical trial has evaluated a home-based rehabilitation program, an approach that could have advantages over inpatient programs and comparable efficacy [12]."

2b) In INTRODUCTION: Specific objectives or hypotheses

Estás editando tu respuesta. Si compartes esta URL, otros usuarios también podrán editar la respuesta.

RELLENAR UNA RESPUESTA NUEVA

Does your paper address CONSORT subitem 2b? *

Copy and paste relevant sections from the manuscript (include quotes in quotation marks "like this" to indicate direct quotes from your manuscript), or elaborate on this item by providing additional information not in the ms, or briefly explain why the item is not applicable/relevant for your study

"The primary objective of the trial was to compare 6-minute walk distances at 7 and 12 weeks after hospital discharge for COVID-19 pneumonia in participants of a combined 6-week home-based respiratory physiotherapy and telephone-based psychological rehabilitation program versus those who received usual care practices."

METHODS

3a) Description of trial design (such as parallel, factorial) including allocation ratio

Does your paper address CONSORT subitem 3a? *

Copy and paste relevant sections from the manuscript (include quotes in quotation marks "like this" to indicate direct quotes from your manuscript), or elaborate on this item by providing additional information not in the ms, or briefly explain why the item is not applicable/relevant for your study

"WAYRA (air in Quechua) was a randomized, controlled, open-label, two-arm clinical trial that evaluated the efficacy of a six-week home-based respiratory physiotherapy and telephone-based psychological program compared to conventional post-hospitalization care, aimed at improving exercise tolerance, lung function, mental health, and QoL outcomes. A baseline assessment was conducted at hospital discharge, with two follow-ups at week 7 and 12 (Figure 1). Due to the nature of the rehabilitation interventions, blinding of participants and the researchers was not possible; however, the data scientist conducted the data analysis was blinded. We used a blinded list pre-uploaded in REDcap [17] which was created by a data administrator who was not involved in study recruitment. To achieve balance in the number of participants assigned to each study arm, the treatment arm was randomized in a 1:1 ratio using permuted blocks of variable size, randomly varying from 2, 4 and 6 participants. The blocks were randomized using Stata v16.0's ralloc command (StataCorp, College Station, TX, 2016)."

3b) Important changes to methods after trial commencement (such as eligibility

Estás editando tu respuesta. Si compartes esta URL, otros usuarios también podrán editar la respuesta. RELLENAR UNA RESPUESTA NUEVA

Does your paper address CONSORT subitem 3b? *

Copy and paste relevant sections from the manuscript (include quotes in quotation marks "like this" to indicate direct quotes from your manuscript), or elaborate on this item by providing additional information not in the ms, or briefly explain why the item is not applicable/relevant for your study

Not applicable.

3b-i) Bug fixes, Downtimes, Content Changes

Bug fixes, Downtimes, Content Changes: ehealth systems are often dynamic systems. A description of changes to methods therefore also includes important changes made on the intervention or comparator during the trial (e.g., major bug fixes or changes in the functionality or content) (5-iii) and other “unexpected events” that may have influenced study design such as staff changes, system failures/downtimes, etc. [2].

	1	2	3	4	5	
subitem not at all important	○	○	○	○	◉	essential

Borrar selección

Does your paper address subitem 3b-i?

Copy and paste relevant sections from the manuscript (include quotes in quotation marks "like this" to indicate direct quotes from your manuscript), or elaborate on this item by providing additional information not in the ms, or briefly explain why the item is not applicable/relevant for your study

Not applicable.

4a) Eligibility criteria for participants

Estás editando tu respuesta. Si compartes esta URL, otros usuarios también podrán editar la respuesta.

RELLENAR UNA RESPUESTA NUEVA

Does your paper address CONSORT subitem 4a? *

Copy and paste relevant sections from the manuscript (include quotes in quotation marks "like this" to indicate direct quotes from your manuscript), or elaborate on this item by providing additional information not in the ms, or briefly explain why the item is not applicable/relevant for your study

"The study population consisted of confirmed cases of COVID-19, diagnosed by serological or molecular tests, including members of both sexes, between 18 and 75 years of age, discharged from hospitalization, who received high flow oxygen (>6 L/min) treatment at any point during hospitalization. Participants with prior respiratory pathology or psychiatric diagnoses were excluded. We list complete eligibility criteria in Table 1"

4a-i) Computer / Internet literacy

Computer / Internet literacy is often an implicit “de facto” eligibility criterion - this should be explicitly clarified.

	1	2	3	4	5	
subitem not at all important	○	○	○	○	●	essential

Borrar selección

Does your paper address subitem 4a-i?

Copy and paste relevant sections from the manuscript (include quotes in quotation marks "like this" to indicate direct quotes from your manuscript), or elaborate on this item by providing additional information not in the ms, or briefly explain why the item is not applicable/relevant for your study

Not applicable.

Estás editando tu respuesta. Si compartes esta URL, otros usuarios también podrán editar la respuesta.

RELLENAR UNA RESPUESTA NUEVA

4a-ii) Open vs. closed, web-based vs. face-to-face assessments:

Open vs. closed, web-based vs. face-to-face assessments: Mention how participants were recruited (online vs. offline), e.g., from an open access website or from a clinic, and clarify if this was a purely web-based trial, or there were face-to-face components (as part of the intervention or for assessment), i.e., to what degree got the study team to know the participant. In online-only trials, clarify if participants were quasi-anonymous and whether having multiple identities was possible or whether technical or logistical measures (e.g., cookies, email confirmation, phone calls) were used to detect/prevent these.

	1	2	3	4	5	
subitem not at all important	○	○	○	○	◉	essential

Borrar selección

Does your paper address subitem 4a-ii? *

Copy and paste relevant sections from the manuscript (include quotes in quotation marks "like this" to indicate direct quotes from your manuscript), or elaborate on this item by providing additional information not in the ms, or briefly explain why the item is not applicable/relevant for your study

Not applicable.

4a-iii) Information giving during recruitment

Information given during recruitment. Specify how participants were briefed for recruitment and in the informed consent procedures (e.g., publish the informed consent documentation as appendix, see also item X26), as this information may have an effect on user self-selection, user expectation and may also bias results.

	1	2	3	4	5	
subitem not at all important	○	○	○	○	◉	essential

Borrar selección

Estás editando tu respuesta. Si compartes esta URL, otros usuarios también podrán editar la respuesta.

RELLENAR UNA RESPUESTA NUEVA

Does your paper address subitem 4a-iii?

Copy and paste relevant sections from the manuscript (include quotes in quotation marks "like this" to indicate direct quotes from your manuscript), or elaborate on this item by providing additional information not in the ms, or briefly explain why the item is not applicable/relevant for your study

"A trained nurse made the initial contact and pre-screening, starting with a daily review of the list of hospitalized patients during the enrollment period, to identify potentially eligible participants among newly discharged patients or those close to discharge in the following days. Potential participants were informed about the study, its procedures and were invited to participate. Those who accepted received a detailed explanation of the nature of the study, randomization, study procedures, their potential risks and benefits, their rights as participants and the study timelines. Potential subjects were explicitly told that participation was not mandatory, that there was no penalty for refusing to participate, and that their clinical treatment at the hospital would not be compromised in any way if they refuse to participate or opt out of the study at any time. All interested patients received the screening informed consent form (Supplemental Material 3) for their review, and they provided a signature once all their questions and concerns were addressed."

4b) Settings and locations where the data were collected

Does your paper address CONSORT subitem 4b? *

Copy and paste relevant sections from the manuscript (include quotes in quotation marks "like this" to indicate direct quotes from your manuscript), or elaborate on this item by providing additional information not in the ms, or briefly explain why the item is not applicable/relevant for your study

"This study was conducted in the COVID-19 ward at Hospital Nacional Cayetano Heredia (HNCH), a public tertiary care hospital and a major referral center that serves approximately 3 million people from underserved neighborhoods in the northern metropolitan area of Lima, Peru's capital. HNCH was one of the main COVID-19 national referral centers throughout the pandemic, with 241 hospitalization beds and 23 ICU beds[14]. During the pandemic, more than half of Lima´s population reported depressive symptoms, with increased rates of symptoms reported among young people with low income and without higher education [15]."

Estás editando tu respuesta. Si compartes esta URL, otros usuarios también podrán editar la respuesta. RELLENAR UNA RESPUESTA NUEVA

4b-i) Report if outcomes were (self-)assessed through online questionnaires

Clearly report if outcomes were (self-)assessed through online questionnaires (as common in web-based trials) or otherwise.

	1	2	3	4	5	
subitem not at all important	○	○	○	○	◉	essential

Borrar selección

Does your paper address subitem 4b-i? *

Copy and paste relevant sections from the manuscript (include quotes in quotation marks "like this" to indicate direct quotes from your manuscript), or elaborate on this item by providing additional information not in the ms, or briefly explain why the item is not applicable/relevant for your study

"For mental health and quality of life assessments, participants responded to questionnaires administered by a trained health worker. We used PHQ-9, GAD-7, and IES-R questionnaires to assess mental health including depression[25], anxiety[26] and post-traumatic stress disorder symptoms [27], respectively. Mental health questionnaires were administered at 2, 4, 7, 8 and 12 weeks after hospitalization by telephone home calls (Figure 1B). We used the SF-36 v2 [28] and EQ-5D [29] questionnaires to measure quality of life at 7 and 12 weeks after discharge. The psychometric properties of the SF-36 questionnaire have been widely studied and consist of 36 items assessing 8 dimensions: physical functioning, physical role, bodily pain, general health, vitality, social functioning, emotional role and mental health[30]. The details of the measurements for the primary and secondary outcomes are summarized in Table 2. We used the Saint George Respiratory Questionnaire to measure respiratory symptoms, an instrument recommended to measuring changes in respiratory health following interventions[31]. We measured respiratory symptoms at 7 and 12 weeks after hospital discharge."

Estás editando tu respuesta. Si compartes esta URL, otros usuarios también podrán editar la respuesta.

RELLENAR UNA RESPUESTA NUEVA

4b-ii) Report how institutional affiliations are displayed

Report how institutional affiliations are displayed to potential participants [on ehealth media], as affiliations with prestigious hospitals or universities may affect volunteer rates, use, and reactions with regards to an intervention.(Not a required item – describe only if this may bias results)

	1	2	3	4	5	
subitem not at all important	○	○	○	○	◉	essential

Borrar selección

Does your paper address subitem 4b-ii?

Copy and paste relevant sections from the manuscript (include quotes in quotation marks "like this" to indicate direct quotes from your manuscript), or elaborate on this item by providing additional information not in the ms, or briefly explain why the item is not applicable/relevant for your study

Not applicable.

5) The interventions for each group with sufficient details to allow replication, including how and when they were actually administered

5-i) Mention names, credential, affiliations of the developers, sponsors, and owners

Mention names, credential, affiliations of the developers, sponsors, and owners [6] (if authors/evaluators are owners or developer of the software, this needs to be declared in a “Conflict of interest” section or mentioned elsewhere in the manuscript).

	1	2	3	4	5	
subitem not at all important	○	○	○	○	◉	essential

Borrar selección

Estás editando tu respuesta. Si compartes esta URL, otros usuarios también podrán editar la respuesta.

RELLENAR UNA RESPUESTA NUEVA

Does your paper address subitem 5-i?

Copy and paste relevant sections from the manuscript (include quotes in quotation marks "like this" to indicate direct quotes from your manuscript), or elaborate on this item by providing additional information not in the ms, or briefly explain why the item is not applicable/relevant for your study

"The authors declare that no competing interests exist."

5-ii) Describe the history/development process

Describe the history/development process of the application and previous formative evaluations (e.g., focus groups, usability testing), as these will have an impact on adoption/use rates and help with interpreting results.

	1	2	3	4	5	
subitem not at all important	○	○	○	○	◉	essential

Borrar selección

Does your paper address subitem 5-ii?

Copy and paste relevant sections from the manuscript (include quotes in quotation marks "like this" to indicate direct quotes from your manuscript), or elaborate on this item by providing additional information not in the ms, or briefly explain why the item is not applicable/relevant for your study

Not applicable.

Estás editando tu respuesta. Si compartes esta URL, otros usuarios también podrán editar la respuesta.

RELLENAR UNA RESPUESTA NUEVA

5-iii) Revisions and updating

Revisions and updating. Clearly mention the date and/or version number of the application/intervention (and comparator, if applicable) evaluated, or describe whether the intervention underwent major changes during the evaluation process, or whether the development and/or content was "frozen" during the trial. Describe dynamic components such as news feeds or changing content which may have an impact on the replicability of the intervention (for unexpected events see item 3b).

	1	2	3	4	5	
subitem not at all important	○	○	○	○	◉	essential

Borrar selección

Does your paper address subitem 5-iii?

Copy and paste relevant sections from the manuscript (include quotes in quotation marks "like this" to indicate direct quotes from your manuscript), or elaborate on this item by providing additional information not in the ms, or briefly explain why the item is not applicable/relevant for your study

Not applicable.

5-iv) Quality assurance methods

Provide information on quality assurance methods to ensure accuracy and quality of information provided [1], if applicable.

	1	2	3	4	5	
subitem not at all important	○	○	○	○	◉	essential

Borrar selección

Estás editando tu respuesta. Si compartes esta URL, otros usuarios también podrán editar la respuesta.

RELLENAR UNA RESPUESTA NUEVA

Does your paper address subitem 5-iv?

Copy and paste relevant sections from the manuscript (include quotes in quotation marks "like this" to indicate direct quotes from your manuscript), or elaborate on this item by providing additional information not in the ms, or briefly explain why the item is not applicable/relevant for your study

Not applicable.

5-v) Ensure replicability by publishing the source code, and/or providing screenshots/screen-capture video, and/or providing flowcharts of the algorithms used

Ensure replicability by publishing the source code, and/or providing screenshots/screen-capture video, and/or providing flowcharts of the algorithms used. Replicability (i.e., other researchers should in principle be able to replicate the study) is a hallmark of scientific reporting.

	1	2	3	4	5	
subitem not at all important	○	○	○	○	◉	essential

Borrar selección

Does your paper address subitem 5-v?

Copy and paste relevant sections from the manuscript (include quotes in quotation marks "like this" to indicate direct quotes from your manuscript), or elaborate on this item by providing additional information not in the ms, or briefly explain why the item is not applicable/relevant for your study

Not applicable.

Estás editando tu respuesta. Si compartes esta URL, otros usuarios también podrán editar la respuesta.

RELLENAR UNA RESPUESTA NUEVA

5-vi) Digital preservation

Digital preservation: Provide the URL of the application, but as the intervention is likely to change or disappear over the course of the years; also make sure the intervention is archived (Internet Archive, webcitation.org, and/or publishing the source code or screenshots/videos alongside the article). As pages behind login screens cannot be archived, consider creating demo pages which are accessible without login.

	1	2	3	4	5	
subitem not at all important	○	○	○	○	◉	essential

Borrar selección

Does your paper address subitem 5-vi?

Copy and paste relevant sections from the manuscript (include quotes in quotation marks "like this" to indicate direct quotes from your manuscript), or elaborate on this item by providing additional information not in the ms, or briefly explain why the item is not applicable/relevant for your study

Not applicable.

5-vii) Access

Access: Describe how participants accessed the application, in what setting/context, if they had to pay (or were paid) or not, whether they had to be a member of specific group. If known, describe how participants obtained “access to the platform and Internet” [1]. To ensure access for editors/reviewers/readers, consider to provide a “backdoor” login account or demo mode for reviewers/readers to explore the application (also important for archiving purposes, see vi).

	1	2	3	4	5	
subitem not at all important	○	○	○	○	◉	essential

Borrar selección

Estás editando tu respuesta. Si compartes esta URL, otros usuarios también podrán editar la respuesta.

RELLENAR UNA RESPUESTA NUEVA

Does your paper address subitem 5-vii? *

Copy and paste relevant sections from the manuscript (include quotes in quotation marks "like this" to indicate direct quotes from your manuscript), or elaborate on this item by providing additional information not in the ms, or briefly explain why the item is not applicable/relevant for your study

Not applicable.

5-viii) Mode of delivery, features/functionalities/components of the intervention and comparator, and the theoretical framework

Describe mode of delivery, features/functionalities/components of the intervention and comparator, and the theoretical framework [6] used to design them (instructional strategy [1], behaviour change techniques, persuasive features, etc., see e.g., [7, 8] for terminology). This includes an in-depth description of the content (including where it is coming from and who developed it) [1]," whether [and how] it is tailored to individual circumstances and allows users to track their progress and receive feedback" [6]. This also includes a description of communication delivery channels and – if computer-mediated communication is a component – whether communication was synchronous or asynchronous [6]. It also includes information on presentation strategies [1], including page design principles, average amount of text on pages, presence of hyperlinks to other resources, etc. [1].

	1	2	3	4	5	
subitem not at all important	○	○	○	○	◉	essential

Borrar selección

Does your paper address subitem 5-viii? *

Copy and paste relevant sections from the manuscript (include quotes in quotation marks "like this" to indicate direct quotes from your manuscript), or elaborate on this item by providing additional information not in the ms, or briefly explain why the item is not applicable/relevant for your study

Not applicable.

Estás editando tu respuesta. Si compartes esta URL, otros usuarios también podrán editar la respuesta.

RELLENAR UNA RESPUESTA NUEVA

5-ix) Describe use parameters

Describe use parameters (e.g., intended “doses” and optimal timing for use). Clarify what instructions or recommendations were given to the user, e.g., regarding timing, frequency, heaviness of use, if any, or was the intervention used ad libitum.

	1	2	3	4	5	
subitem not at all important	○	○	○	○	◉	essential

Borrar selección

Does your paper address subitem 5-ix?

Copy and paste relevant sections from the manuscript (include quotes in quotation marks "like this" to indicate direct quotes from your manuscript), or elaborate on this item by providing additional information not in the ms, or briefly explain why the item is not applicable/relevant for your study

Not applicable.

5-x) Clarify the level of human involvement

Clarify the level of human involvement (care providers or health professionals, also technical assistance) in the e-intervention or as co-intervention (detail number and expertise of professionals involved, if any, as well as “type of assistance offered, the timing and frequency of the support, how it is initiated, and the medium by which the assistance is delivered”. It may be necessary to distinguish between the level of human involvement required for the trial, and the level of human involvement required for a routine application outside of a RCT setting (discuss under item 21 – generalizability).

	1	2	3	4	5	
subitem not at all important	○	○	○	○	◉	essential

Borrar selección

Estás editando tu respuesta. Si compartes esta URL, otros usuarios también podrán editar la respuesta.

RELLENAR UNA RESPUESTA NUEVA

Does your paper address subitem 5-x?

Copy and paste relevant sections from the manuscript (include quotes in quotation marks "like this" to indicate direct quotes from your manuscript), or elaborate on this item by providing additional information not in the ms, or briefly explain why the item is not applicable/relevant for your study

Not applicable.

5-xi) Report any prompts/reminders used

Report any prompts/reminders used: Clarify if there were prompts (letters, emails, phone calls, SMS) to use the application, what triggered them, frequency etc. It may be necessary to distinguish between the level of prompts/reminders required for the trial, and the level of prompts/reminders for a routine application outside of a RCT setting (discuss under item 21 – generalizability).

	1	2	3	4	5	
subitem not at all important	○	○	○	○	◉	essential

Borrar selección

Does your paper address subitem 5-xi? *

Copy and paste relevant sections from the manuscript (include quotes in quotation marks "like this" to indicate direct quotes from your manuscript), or elaborate on this item by providing additional information not in the ms, or briefly explain why the item is not applicable/relevant for your study

Not applicable.

Estás editando tu respuesta. Si compartes esta URL, otros usuarios también podrán editar la respuesta. RELLENAR UNA RESPUESTA NUEVA

5-xii) Describe any co-interventions (incl. training/support)

Describe any co-interventions (incl. training/support): Clearly state any interventions that are provided in addition to the targeted eHealth intervention, as ehealth intervention may not be designed as stand-alone intervention. This includes training sessions and support [1]. It may be necessary to distinguish between the level of training required for the trial, and the level of training for a routine application outside of a RCT setting (discuss under item 21 – generalizability.

	1	2	3	4	5	
subitem not at all important	○	○	○	○	◉	essential

Borrar selección

Does your paper address subitem 5-xii? *

Copy and paste relevant sections from the manuscript (include quotes in quotation marks "like this" to indicate direct quotes from your manuscript), or elaborate on this item by providing additional information not in the ms, or briefly explain why the item is not applicable/relevant for your study

Not applicable.

6a) Completely defined pre-specified primary and secondary outcome measures, including how and when they were assessed

Estás editando tu respuesta. Si compartes esta URL, otros usuarios también podrán editar la respuesta. RELLENAR UNA RESPUESTA NUEVA

Does your paper address CONSORT subitem 6a? *

Copy and paste relevant sections from the manuscript (include quotes in quotation marks "like this" to indicate direct quotes from your manuscript), or elaborate on this item by providing additional information not in the ms, or briefly explain why the item is not applicable/relevant for your study

"The primary health outcome was the change in six-minute walk distance (6MWD) at 7 and 12 weeks after discharge compared to baseline (Figure 1B). The six-minute walk test (6MWT) is a sub-maximal effort test of cardiorespiratory functional capacity with correlation to quality of life and independence in activities of daily living[20]. The 6MWT is widely used for the follow-up of patients after hospitalization because patients can self-regulate the intensity of the activity, resting as often as desired, leading to minimal risks of adverse events during the activity [20]. The test was performed in a 20-meter hospital aisle marked according to the recommendations of the American Thoracic Society [21,22]. Participants who demonstrated instability while standing, generalized weakness, or those with a decrease in oxygen saturation below 90% or increase heart rate >130 beats per minute while standing did not perform the test. In this case, those participants were considered to have covered 0 meters. Each participant only attempted the test once. The number of meters walked in the 6MWT was calculated by the assessor and recorded in the data collection form. In Figure 1B, we show the outcome assessment schedule."
"A trained physician measured forced expiratory volume in the first second (FEV1), forced vital capacity (FVC) and FEV1/FVC ratio using a portable spirometer (Easy-On-PC, ndd, Zurich, Switzerland). These spirometers are frequently used in pulmonary research because their calibration remains stable over time [23]. We aimed to obtain at least three acceptable and two reproducible tests following joint recommendations of the American Thoracic Society and European Respiratory Societies[24] or until the participant was no longer able to tolerate the procedure. Results were recorded on a secure interface (EasyOnConnect, ndd, Zurich, Switzerland) on a personal computer."
"For mental health and quality of life assessments, participants responded to questionnaires administered by a trained health worker. We used PHQ-9, GAD-7, and IES-R questionnaires to assess mental health including depression[25], anxiety[26] and post-traumatic stress disorder symptoms [27], respectively. Mental health questionnaires were administered at 2, 4, 7, 8 and 12 weeks after hospitalization by telephone home calls (Figure 1B). We used the SF-36 v2 [28] and EQ-5D [29] questionnaires to measure quality of life at 7 and 12 weeks after discharge. The psychometric properties of the SF-36 questionnaire have been widely studied and consist of 36 items assessing 8 dimensions: physical functioning, physical role, bodily pain, general health, vitality, social functioning, emotional role and mental health[30]. The details of the measurements for the primary and secondary outcomes are summarized in Table 2. We used the Saint George Respiratory Questionnaire to measure respiratory symptoms, an instrument recommended to measuring changes in respiratory health following interventions[31]. We measured respiratory symptoms at 7 and 12 weeks after hospital discharge."

Estás editando tu respuesta. Si compartes esta URL, otros usuarios también podrán editar la respuesta.

RELLENAR UNA RESPUESTA NUEVA

6a-i) Online questionnaires: describe if they were validated for online use and apply CHERRIES items to describe how the questionnaires were designed/deployed

If outcomes were obtained through online questionnaires, describe if they were validated for online use and apply CHERRIES items to describe how the questionnaires were designed/deployed [9].

	1	2	3	4	5	
subitem not at all important	○	○	○	○	◉	essential

Borrar selección

Does your paper address subitem 6a-i?

Copy and paste relevant sections from manuscript text

Not applicable.

6a-ii) Describe whether and how "use" (including intensity of use/dosage) was defined/measured/monitored

Describe whether and how "use" (including intensity of use/dosage) was defined/measured/monitored (logins, logfile analysis, etc.). Use/adoption metrics are important process outcomes that should be reported in any ehealth trial.

	1	2	3	4	5	
subitem not at all important	○	○	○	○	◉	essential

Borrar selección

Does your paper address subitem 6a-ii?

Copy and paste relevant sections from manuscript text

Not applicable.

Estás editando tu respuesta. Si compartes esta URL, otros usuarios también podrán editar la respuesta.

RELLENAR UNA RESPUESTA NUEVA

6a-iii) Describe whether, how, and when qualitative feedback from participants was obtained

Describe whether, how, and when qualitative feedback from participants was obtained (e.g., through emails, feedback forms, interviews, focus groups).

	1	2	3	4	5	
subitem not at all important	○	○	○	○	◉	essential

Borrar selección

Does your paper address subitem 6a-iii?

Copy and paste relevant sections from manuscript text

Not applicable.

6b) Any changes to trial outcomes after the trial commenced, with reasons

Does your paper address CONSORT subitem 6b? *

Copy and paste relevant sections from the manuscript (include quotes in quotation marks "like this" to indicate direct quotes from your manuscript), or elaborate on this item by providing additional information not in the ms, or briefly explain why the item is not applicable/relevant for your study

Not applicable.

7a) How sample size was determined

NPT: When applicable, details of whether and how the clustering by care provides or centers was addressed

Estás editando tu respuesta. Si compartes esta URL, otros usuarios también podrán editar la respuesta.

RELLENAR UNA RESPUESTA NUEVA

7a-i) Describe whether and how expected attrition was taken into account when calculating the sample size

Describe whether and how expected attrition was taken into account when calculating the sample size.

	1	2	3	4	5	
subitem not at all important	○	○	○	○	◉	essential

Borrar selección

Does your paper address subitem 7a-i?

Copy and paste relevant sections from manuscript title (include quotes in quotation marks "like this" to indicate direct quotes from your manuscript), or elaborate on this item by providing additional information not in the ms, or briefly explain why the item is not applicable/relevant for your study

"We used STATA Version 16 (StataCorp, Texas, USA) to calculate the ideal sample size. We attempted to enroll 108 participants in total, allocating 54 to each treatment arm. We estimate that a sample size of 86 participants would be able to detect a mean difference between arms of 55.1 meters in 6MWD according to the study by Liu et al [11] considering a 95% confidence level and 90% power. Assuming a potential 20% rate of rejection or loss to follow-up, we aim to achieve a sample of 108 participants."

7b) When applicable, explanation of any interim analyses and stopping guidelines

Does your paper address CONSORT subitem 7b? *

Copy and paste relevant sections from the manuscript (include quotes in quotation marks "like this" to indicate direct quotes from your manuscript), or elaborate on this item by providing additional information not in the ms, or briefly explain why the item is not applicable/relevant for your study

"There will be no formal interim analysis of the data."

Estás editando tu respuesta. Si compartes esta URL, otros usuarios también podrán editar la respuesta.

RELLENAR UNA RESPUESTA NUEVA

Does your paper address CONSORT subitem 8a? *

Copy and paste relevant sections from the manuscript (include quotes in quotation marks "like this" to indicate direct quotes from your manuscript), or elaborate on this item by providing additional information not in the ms, or briefly explain why the item is not applicable/relevant for your study

"We used a blinded list pre-uploaded in REDcap [17] which was created by a data administrator who was not involved in study recruitment. To achieve balance in the number of participants assigned to each study arm, the treatment arm was randomized in a 1:1 ratio using permuted blocks of variable size, randomly varying from 2, 4 and 6 participants. The blocks were randomized using Stata v16.0's ralloc command (StataCorp, College Station, TX, 2016). Finally, all participants were scheduled for follow-up visits as described in the Figure 1B. Sample selection and enrolment took place between the day before hospital discharge and up to three days after discharge."

8b) Type of randomisation; details of any restriction (such as blocking and block size)

Does your paper address CONSORT subitem 8b? *

Copy and paste relevant sections from the manuscript (include quotes in quotation marks "like this" to indicate direct quotes from your manuscript), or elaborate on this item by providing additional information not in the ms, or briefly explain why the item is not applicable/relevant for your study

"We used a blinded list pre-uploaded in REDcap [17] which was created by a data administrator who was not involved in study recruitment. To achieve balance in the number of participants assigned to each study arm, the treatment arm was randomized in a 1:1 ratio using permuted blocks of variable size, randomly varying from 2, 4 and 6 participants. The blocks were randomized using Stata v16.0's ralloc command (StataCorp, College Station, TX, 2016). Finally, all participants were scheduled for follow-up visits as described in the Figure 1B. Sample selection and enrolment took place between the day before hospital discharge and up to three days after discharge."

9) Mechanism used to implement the random allocation sequence (such as sequentially numbered containers), describing any steps taken to conceal the sequence until interventions were assigned

Estás editando tu respuesta. Si compartes esta URL, otros usuarios también podrán editar la respuesta. RELLENAR UNA RESPUESTA NUEVA

Does your paper address CONSORT subitem 9? *

Copy and paste relevant sections from the manuscript (include quotes in quotation marks "like this" to indicate direct quotes from your manuscript), or elaborate on this item by providing additional information not in the ms, or briefly explain why the item is not applicable/relevant for your study

"We used a blinded list pre-uploaded in REDcap [17] which was created by a data administrator who was not involved in study recruitment. To achieve balance in the number of participants assigned to each study arm, the treatment arm was randomized in a 1:1 ratio using permuted blocks of variable size, randomly varying from 2, 4 and 6 participants. The blocks were randomized using Stata v16.0's ralloc command (StataCorp, College Station, TX, 2016). Finally, all participants were scheduled for follow-up visits as described in the Figure 1B. Sample selection and enrolment took place between the day before hospital discharge and up to three days after discharge."

10) Who generated the random allocation sequence, who enrolled participants, and who assigned participants to interventions

Does your paper address CONSORT subitem 10? *

Copy and paste relevant sections from the manuscript (include quotes in quotation marks "like this" to indicate direct quotes from your manuscript), or elaborate on this item by providing additional information not in the ms, or briefly explain why the item is not applicable/relevant for your study

"We used a blinded list pre-uploaded in REDcap [17] which was created by a data administrator who was not involved in study recruitment."

11a) If done, who was blinded after assignment to interventions (for example, participants, care providers, those assessing outcomes) and how

NPT: Whether or not administering co-interventions were blinded to group assignment

Estás editando tu respuesta. Si compartes esta URL, otros usuarios también podrán editar la respuesta.

RELLENAR UNA RESPUESTA NUEVA

11a-i) Specify who was blinded, and who wasn't

Specify who was blinded, and who wasn't. Usually, in web-based trials it is not possible to blind the participants [1, 3] (this should be clearly acknowledged), but it may be possible to blind outcome assessors, those doing data analysis or those administering co-interventions (if any).

	1	2	3	4	5	
subitem not at all important	○	○	○	○	◉	essential

Borrar selección

Does your paper address subitem 11a-i? *

Copy and paste relevant sections from the manuscript (include quotes in quotation marks "like this" to indicate direct quotes from your manuscript), or elaborate on this item by providing additional information not in the ms, or briefly explain why the item is not applicable/relevant for your study

"Due to the nature of the rehabilitation interventions, blinding of participants and the researchers was not possible; however, the data scientist conducted the data analysis was blinded."

11a-ii) Discuss e.g., whether participants knew which intervention was the "intervention of interest" and which one was the "comparator"

Informed consent procedures (4a-ii) can create biases and certain expectations - discuss e.g., whether participants knew which intervention was the "intervention of interest" and which one was the "comparator".

	1	2	3	4	5	
subitem not at all important	○	○	○	○	◉	essential

Borrar selección

Estás editando tu respuesta. Si compartes esta URL, otros usuarios también podrán editar la respuesta.

RELLENAR UNA RESPUESTA NUEVA

Does your paper address subitem 11a-ii?

Copy and paste relevant sections from the manuscript (include quotes in quotation marks "like this" to indicate direct quotes from your manuscript), or elaborate on this item by providing additional information not in the ms, or briefly explain why the item is not applicable/relevant for your study

Same answer as above

11b) If relevant, description of the similarity of interventions

(this item is usually not relevant for ehealth trials as it refers to similarity of a placebo or sham intervention to a active medication/intervention)

Does your paper address CONSORT subitem 11b? *

Copy and paste relevant sections from the manuscript (include quotes in quotation marks "like this" to indicate direct quotes from your manuscript), or elaborate on this item by providing additional information not in the ms, or briefly explain why the item is not applicable/relevant for your study

Not applicable.

12a) Statistical methods used to compare groups for primary and secondary outcomes

NPT: When applicable, details of whether and how the clustering by care providers or centers was addressed

Estás editando tu respuesta. Si compartes esta URL, otros usuarios también podrán editar la respuesta.

RELLENAR UNA RESPUESTA NUEVA

Does your paper address CONSORT subitem 12a? *

Copy and paste relevant sections from the manuscript (include quotes in quotation marks "like this" to indicate direct quotes from your manuscript), or elaborate on this item by providing additional information not in the ms, or briefly explain why the item is not applicable/relevant for your study

"We will conduct an intention-to-treat analysis of primary and secondary outcomes at 7- and 12-weeks after hospital discharge. The final analysis will be performed when all 108 participants completed the trial. We will compare the 6MWD (meters), FVC (liters), FEV1 (liters) and questionnaire scores at 7 and 12 weeks after hospitalization discharge between intervention and control arms. In the secondary analyses, we will compare the proportion of participants with restrictive spirometry pattern, depression, anxiety, and PSTD at 7- and 12-weeks after hospital discharge between intervention and control arms. In addition, following the recommendation by Cocks and Torgerson, we will use a one-sided 80% confidence interval to determine if this trial should proceed to a phase III trial [32]. As a sensitivity analysis, the association between the number of therapies received and outcomes will be examined, adjusting for potential confounders that will be identify by a causal acyclic diagram. There will be no formal interim analysis of the data."

12a-i) Imputation techniques to deal with attrition / missing values

Imputation techniques to deal with attrition / missing values: Not all participants will use the intervention/comparator as intended and attrition is typically high in ehealth trials. Specify how participants who did not use the application or dropped out from the trial were treated in the statistical analysis (a complete case analysis is strongly discouraged, and simple imputation techniques such as LOCF may also be problematic [4]).

	1	2	3	4	5	
subitem not at all important	○	○	○	○	◉	essential

Borrar selección

Does your paper address subitem 12a-i? *

Copy and paste relevant sections from the manuscript (include quotes in quotation marks "like this" to indicate direct quotes from your manuscript), or elaborate on this item by providing additional information not in the ms, or briefly explain why the item is not applicable/relevant for your study

Not applicable.

Estás editando tu respuesta. Si compartes esta URL, otros usuarios también podrán editar la respuesta.

RELLENAR UNA RESPUESTA NUEVA

12b) Methods for additional analyses, such as subgroup analyses and adjusted analyses

Does your paper address CONSORT subitem 12b? *

Copy and paste relevant sections from the manuscript (include quotes in quotation marks "like this" to indicate direct quotes from your manuscript), or elaborate on this item by providing additional information not in the ms, or briefly explain why the item is not applicable/relevant for your study

"As a sensitivity analysis, the association between the number of therapies received and outcomes will be examined, adjusting for potential confounders that will be identify by a causal acyclic diagram."

X26) REB/IRB Approval and Ethical Considerations [recommended as subheading under "Methods"] (not a CONSORT item)

X26-i) Comment on ethics committee approval

	1	2	3	4	5	
subitem not at all important	○	○	○	○	◉	essential

Borrar selección

Does your paper address subitem X26-i?

Copy and paste relevant sections from the manuscript (include quotes in quotation marks "like this" to indicate direct quotes from your manuscript), or elaborate on this item by providing additional information not in the ms, or briefly explain why the item is not applicable/relevant for your study

"Ref # 202852. Institutional Review Board, Universidad Cayetano Heredia, Lima, Peru
Ref # 085-2020. Institutional Review Board, Hospital Nacional Cayetano Heredia, Lima, Peru"

Estás editando tu respuesta. Si compartes esta URL, otros usuarios también podrán editar la respuesta.

RELLENAR UNA RESPUESTA NUEVA

x26-ii) Outline informed consent procedures

Outline informed consent procedures e.g., if consent was obtained offline or online (how? Checkbox, etc.?), and what information was provided (see 4a-ii). See [6] for some items to be included in informed consent documents.

	1	2	3	4	5	
subitem not at all important	○	○	○	○	◉	essential

Borrar selección

Does your paper address subitem X26-ii?

Copy and paste relevant sections from the manuscript (include quotes in quotation marks "like this" to indicate direct quotes from your manuscript), or elaborate on this item by providing additional information not in the ms, or briefly explain why the item is not applicable/relevant for your study

"A trained nurse made the initial contact and pre-screening, starting with a daily review of the list of hospitalized patients during the enrollment period, to identify potentially eligible participants among newly discharged patients or those close to discharge in the following days. Potential participants were informed about the study, its procedures and were invited to participate. Those who accepted received a detailed explanation of the nature of the study, randomization, study procedures, their potential risks and benefits, their rights as participants and the study timelines. Potential subjects were explicitly told that participation was not mandatory, that there was no penalty for refusing to participate, and that their clinical treatment at the hospital would not be compromised in any way if they refuse to participate or opt out of the study at any time. All interested patients received the screening informed consent form (Supplemental Material 3) for their review, and they provided a signature once all their questions and concerns were addressed."

X26-iii) Safety and security procedures

Safety and security procedures, incl. privacy considerations, and any steps taken to reduce the likelihood or detection of harm (e.g., education and training, availability of a hotline)

	1	2	3	4	5	
subitem not at all important	○	○	○	○	◉	essential

Estás editando tu respuesta. Si compartes esta URL, otros usuarios también podrán editar la respuesta.

RELLENAR UNA RESPUESTA NUEVA

Does your paper address subitem X26-iii?

Copy and paste relevant sections from the manuscript (include quotes in quotation marks "like this" to indicate direct quotes from your manuscript), or elaborate on this item by providing additional information not in the ms, or briefly explain why the item is not applicable/relevant for your study

"To protect participant's confidentiality, all collected data of clinical assessments were linked to a unique ID code. Data were registered in paper form and electronically. The electronic information was stored, backed-up and secured by password protection in the RedCap server. Paper forms were archived in secure locked cabinets in the HNCH. All confidential information, including participant's contact details or other sensible data, was only accessible to authorized staff from the project. Long-term data management complied with UPCH research policy."

"Both control and intervention arm participants benefited from the study. These benefits included education and counseling on respiratory disease risk factors, reporting of pulmonary function test results by a specialist physician, and referral to trained personnel if necessary."

"Potential risks of respiratory rehabilitation were oxygen desaturation, palpitations, sweating, arrhythmias, chest tightness, shortness of breath, and muscle aches. Potential risks of psychological support were symptom substitution, dependence on the physiotherapist, stigmatization, problems in social relationships or even separation, as well as alcohol or drug abuse, deliberate self-harm and suicidal ideation or attempts. Adverse events of the interventions were collected after subjects gave informed consent and were duly enrolled in the study. If adverse events were detected, the field staff immediately notified the study coordinator and the management protocol was activated."

RESULTS

13a) For each group, the numbers of participants who were randomly assigned, received intended treatment, and were analysed for the primary outcome

NPT: The number of care providers or centers performing the intervention in each group and the number of patients treated by each care provider in each center

Estás editando tu respuesta. Si compartes esta URL, otros usuarios también podrán editar la respuesta. RELLENAR UNA RESPUESTA NUEVA

Does your paper address CONSORT subitem 13a? *

Copy and paste relevant sections from the manuscript (include quotes in quotation marks "like this" to indicate direct quotes from your manuscript), or elaborate on this item by providing additional information not in the ms, or briefly explain why the item is not applicable/relevant for your study

Not applicable.

13b) For each group, losses and exclusions after randomisation, together with reasons

Does your paper address CONSORT subitem 13b? (NOTE: Preferably, this is shown in a CONSORT flow diagram) *

Copy and paste relevant sections from the manuscript (include quotes in quotation marks "like this" to indicate direct quotes from your manuscript), or elaborate on this item by providing additional information not in the ms, or briefly explain why the item is not applicable/relevant for your study

Not applicable.

13b-i) Attrition diagram

Strongly recommended: An attrition diagram (e.g., proportion of participants still logging in or using the intervention/comparator in each group plotted over time, similar to a survival curve) or other figures or tables demonstrating usage/dose/engagement.

	1	2	3	4	5	
subitem not at all important	○	○	○	○	●	essential

Borrar selección

Estás editando tu respuesta. Si compartes esta URL, otros usuarios también podrán editar la respuesta. RELLENAR UNA RESPUESTA NUEVA

Does your paper address subitem 13b-i?

Copy and paste relevant sections from the manuscript or cite the figure number if applicable (include quotes in quotation marks "like this" to indicate direct quotes from your manuscript), or elaborate on this item by providing additional information not in the ms, or briefly explain why the item is not applicable/relevant for your study

Not applicable.

14a) Dates defining the periods of recruitment and follow-up

Does your paper address CONSORT subitem 14a? *

Copy and paste relevant sections from the manuscript (include quotes in quotation marks "like this" to indicate direct quotes from your manuscript), or elaborate on this item by providing additional information not in the ms, or briefly explain why the item is not applicable/relevant for your study

"The trial, which obtained funding in July 2020, was approved by the Institutional Review Board in September 2020. Data collection began in October 2020. Enrollment of participants began in October 2021 and was completed in June 2021. An amendment was submitted, and ethics approval was obtained. Data analysis is still ongoing. Results of the study are expected to be published by the end of 2022."

14a-i) Indicate if critical “secular events” fell into the study period

Indicate if critical “secular events” fell into the study period, e.g., significant changes in Internet resources available or “changes in computer hardware or Internet delivery resources”

	1	2	3	4	5	
subitem not at all important	○	○	○	○	◉	essential

Borrar selección

Estás editando tu respuesta. Si compartes esta URL, otros usuarios también podrán editar la respuesta.

RELLENAR UNA RESPUESTA NUEVA

Does your paper address subitem 14a-i?

Copy and paste relevant sections from the manuscript (include quotes in quotation marks "like this" to indicate direct quotes from your manuscript), or elaborate on this item by providing additional information not in the ms, or briefly explain why the item is not applicable/relevant for your study

Not applicable.

14b) Why the trial ended or was stopped (early)

Does your paper address CONSORT subitem 14b? *

Copy and paste relevant sections from the manuscript (include quotes in quotation marks "like this" to indicate direct quotes from your manuscript), or elaborate on this item by providing additional information not in the ms, or briefly explain why the item is not applicable/relevant for your study

The trial ended within the intended time period

15) A table showing baseline demographic and clinical characteristics for each group

NPT: When applicable, a description of care providers (case volume, qualification, expertise, etc.) and centers (volume) in each group

Does your paper address CONSORT subitem 15? *

Copy and paste relevant sections from the manuscript (include quotes in quotation marks "like this" to indicate direct quotes from your manuscript), or elaborate on this item by providing additional information not in the ms, or briefly explain why the item is not applicable/relevant for your study

Not applicable.

Estás editando tu respuesta. Si compartes esta URL, otros usuarios también podrán editar la respuesta. RELLENAR UNA RESPUESTA NUEVA

15-i) Report demographics associated with digital divide issues

In ehealth trials it is particularly important to report demographics associated with digital divide issues, such as age, education, gender, social-economic status, computer/Internet/ehealth literacy of the participants, if known.

	1	2	3	4	5	
subitem not at all important	○	○	○	○	◉	essential

Borrar selección

Does your paper address subitem 15-i? *

Copy and paste relevant sections from the manuscript (include quotes in quotation marks "like this" to indicate direct quotes from your manuscript), or elaborate on this item by providing additional information not in the ms, or briefly explain why the item is not applicable/relevant for your study

Not applicable.

16) For each group, number of participants (denominator) included in each analysis and whether the analysis was by original assigned groups

16-i) Report multiple “denominators” and provide definitions

Report multiple “denominators” and provide definitions: Report N’s (and effect sizes) “across a range of study participation [and use] thresholds” [1], e.g., N exposed, N consented, N used more than x times, N used more than y weeks, N participants “used” the intervention/comparator at specific pre-defined time points of interest (in absolute and relative numbers per group). Always clearly define “use” of the intervention.

	1	2	3	4	5	
subitem not at all important	○	○	○	○	◉	essential

Borrar selección

Estás editando tu respuesta. Si compartes esta URL, otros usuarios también podrán editar la respuesta.

RELLENAR UNA RESPUESTA NUEVA

Does your paper address subitem 16-i? *

Copy and paste relevant sections from the manuscript (include quotes in quotation marks "like this" to indicate direct quotes from your manuscript), or elaborate on this item by providing additional information not in the ms, or briefly explain why the item is not applicable/relevant for your study

Not applicable.

16-ii) Primary analysis should be intent-to-treat

Primary analysis should be intent-to-treat, secondary analyses could include comparing only “users”, with the appropriate caveats that this is no longer a randomized sample (see 18-i).

	1	2	3	4	5	
subitem not at all important	○	○	○	○	◉	essential

Borrar selección

Does your paper address subitem 16-ii?

Copy and paste relevant sections from the manuscript (include quotes in quotation marks "like this" to indicate direct quotes from your manuscript), or elaborate on this item by providing additional information not in the ms, or briefly explain why the item is not applicable/relevant for your study

Not applicable.

17a) For each primary and secondary outcome, results for each group, and the estimated effect size and its precision (such as 95% confidence interval)

Estás editando tu respuesta. Si compartes esta URL, otros usuarios también podrán editar la respuesta. RELLENAR UNA RESPUESTA NUEVA

Does your paper address CONSORT subitem 17a? *

Copy and paste relevant sections from the manuscript (include quotes in quotation marks "like this" to indicate direct quotes from your manuscript), or elaborate on this item by providing additional information not in the ms, or briefly explain why the item is not applicable/relevant for your study

Not applicable.

17a-i) Presentation of process outcomes such as metrics of use and intensity of use

In addition to primary/secondary (clinical) outcomes, the presentation of process outcomes such as metrics of use and intensity of use (dose, exposure) and their operational definitions is critical. This does not only refer to metrics of attrition (13-b) (often a binary variable), but also to more continuous exposure metrics such as "average session length". These must be accompanied by a technical description how a metric like a "session" is defined (e.g., timeout after idle time) [1] (report under item 6a).

	1	2	3	4	5	
subitem not at all important	○	○	○	○	◉	essential

Borrar selección

Does your paper address subitem 17a-i?

Copy and paste relevant sections from the manuscript (include quotes in quotation marks "like this" to indicate direct quotes from your manuscript), or elaborate on this item by providing additional information not in the ms, or briefly explain why the item is not applicable/relevant for your study

Not applicable.

17b) For binary outcomes, presentation of both absolute and relative effect sizes is recommended

Estás editando tu respuesta. Si compartes esta URL, otros usuarios también podrán editar la respuesta. RELLENAR UNA RESPUESTA NUEVA

Does your paper address CONSORT subitem 17b? *

Copy and paste relevant sections from the manuscript (include quotes in quotation marks "like this" to indicate direct quotes from your manuscript), or elaborate on this item by providing additional information not in the ms, or briefly explain why the item is not applicable/relevant for your study

Not applicable.

18) Results of any other analyses performed, including subgroup analyses and adjusted analyses, distinguishing pre-specified from exploratory

Does your paper address CONSORT subitem 18? *

Copy and paste relevant sections from the manuscript (include quotes in quotation marks "like this" to indicate direct quotes from your manuscript), or elaborate on this item by providing additional information not in the ms, or briefly explain why the item is not applicable/relevant for your study

Not applicable.

18-i) Subgroup analysis of comparing only users

A subgroup analysis of comparing only users is not uncommon in ehealth trials, but if done, it must be stressed that this is a self-selected sample and no longer an unbiased sample from a randomized trial (see 16-iii).

	1	2	3	4	5	
subitem not at all important	○	○	○	○	◉	essential

Borrar selección

Estás editando tu respuesta. Si compartes esta URL, otros usuarios también podrán editar la respuesta.

RELLENAR UNA RESPUESTA NUEVA

Does your paper address subitem 18-i?

Copy and paste relevant sections from the manuscript (include quotes in quotation marks "like this" to indicate direct quotes from your manuscript), or elaborate on this item by providing additional information not in the ms, or briefly explain why the item is not applicable/relevant for your study

Not applicable.

19) All important harms or unintended effects in each group
(for specific guidance see CONSORT for harms)

Does your paper address CONSORT subitem 19? *

Copy and paste relevant sections from the manuscript (include quotes in quotation marks "like this" to indicate direct quotes from your manuscript), or elaborate on this item by providing additional information not in the ms, or briefly explain why the item is not applicable/relevant for your study

Not applicable.

19-i) Include privacy breaches, technical problems

Include privacy breaches, technical problems. This does not only include physical “harm” to participants, but also incidents such as perceived or real privacy breaches [1], technical problems, and other unexpected/unintended incidents. “Unintended effects” also includes unintended positive effects [2].

	1	2	3	4	5	
subitem not at all important	○	○	○	○	◉	essential

Borrar selección

Estás editando tu respuesta. Si compartes esta URL, otros usuarios también podrán editar la respuesta.

RELLENAR UNA RESPUESTA NUEVA

Does your paper address subitem 19-i?

Copy and paste relevant sections from the manuscript (include quotes in quotation marks "like this" to indicate direct quotes from your manuscript), or elaborate on this item by providing additional information not in the ms, or briefly explain why the item is not applicable/relevant for your study

Not applicable.

19-ii) Include qualitative feedback from participants or observations from staff/researchers

Include qualitative feedback from participants or observations from staff/researchers, if available, on strengths and shortcomings of the application, especially if they point to unintended/unexpected effects or uses. This includes (if available) reasons for why people did or did not use the application as intended by the developers.

	1	2	3	4	5	
subitem not at all important	○	○	○	○	◉	essential

Borrar selección

Does your paper address subitem 19-ii?

Copy and paste relevant sections from the manuscript (include quotes in quotation marks "like this" to indicate direct quotes from your manuscript), or elaborate on this item by providing additional information not in the ms, or briefly explain why the item is not applicable/relevant for your study

Not applicable.

DISCUSSION

22) Interpretation consistent with results, balancing benefits and harms, and considering other relevant evidence

Estás editando tu respuesta. Si compartes esta URL, otros usuarios también podrán editar la respuesta.

RELLENAR UNA RESPUESTA NUEVA

22-i) Restate study questions and summarize the answers suggested by the data, starting with primary outcomes and process outcomes (use)

Restate study questions and summarize the answers suggested by the data, starting with primary outcomes and process outcomes (use).

	1	2	3	4	5	
subitem not at all important	○	○	○	○	◉	essential

Borrar selección

Does your paper address subitem 22-i? *

Copy and paste relevant sections from the manuscript (include quotes in quotation marks "like this" to indicate direct quotes from your manuscript), or elaborate on this item by providing additional information not in the ms, or briefly explain why the item is not applicable/relevant for your study

"WAYRA is the first randomized controlled trial evaluating integrated pulmonary and mental health rehabilitation for hospitalized COVID-19 survivors in a low- and middle-income country setting. Through this trial we gained experience in implementing a remote rehabilitation program in a resource-limited country and generated evidence on pulmonary, mental health and quality of life alterations following COVID-19 the first three months after hospitalization. We hypothesize that the participation on the combined program will improve pulmonary, physical, and mental health outcomes. We hope that our study will provide a reliable basis for further clinical trials focused on comprehensive rehabilitation in patients hospitalized for severe COVID-19 and other lung diseases."

22-ii) Highlight unanswered new questions, suggest future research

Highlight unanswered new questions, suggest future research.

	1	2	3	4	5	
subitem not at all important	○	○	○	○	◉	essential

Borrar selección

Estás editando tu respuesta. Si compartes esta URL, otros usuarios también podrán editar la respuesta. RELLENAR UNA RESPUESTA NUEVA

Does your paper address subitem 22-ii?

Copy and paste relevant sections from the manuscript (include quotes in quotation marks "like this" to indicate direct quotes from your manuscript), or elaborate on this item by providing additional information not in the ms, or briefly explain why the item is not applicable/relevant for your study

"The burden of long-term complications of COVID-19 will increase because, although several countries have achieved vaccination coverage, newly emerging variants are resistant to vaccines [33]. The most frequently affected systems are respiratory and physical, but psychological symptoms are also common [3]. Despite this, there are still few published studies that propose strategies for COVID-19 survivors with long-term complications, and far fewer that include mental health. There are approximately 200 trials registered with ClinicalTrials.gov evaluating rehabilitation programs for COVID-19 survivors. Most include pulmonary rehabilitation, but only a few have a psychological component [34]."

20) Trial limitations, addressing sources of potential bias, imprecision, and, if relevant, multiplicity of analyses

20-i) Typical limitations in ehealth trials

Typical limitations in ehealth trials: Participants in ehealth trials are rarely blinded. Ehealth trials often look at a multiplicity of outcomes, increasing risk for a Type I error. Discuss biases due to non-use of the intervention/usability issues, biases through informed consent procedures, unexpected events.

	1	2	3	4	5	
subitem not at all important	○	○	○	○	◉	essential

Borrar selección

Estás editando tu respuesta. Si compartes esta URL, otros usuarios también podrán editar la respuesta.

RELLENAR UNA RESPUESTA NUEVA

Does your paper address subitem 20-i? *

Copy and paste relevant sections from the manuscript (include quotes in quotation marks "like this" to indicate direct quotes from your manuscript), or elaborate on this item by providing additional information not in the ms, or briefly explain why the item is not applicable/relevant for your study

"The main strength of this study is that the calculated sample size has the power to detect a clinically significant difference between the groups. On the other hand, this study has some limitations to be considered. First, mental health and quality of life outcomes will be assessed with screening tools. Second, this trial has an open-label design due the nature of intervention."

21) Generalisability (external validity, applicability) of the trial findings

NPT: External validity of the trial findings according to the intervention, comparators, patients, and care providers or centers involved in the trial

21-i) Generalizability to other populations

Generalizability to other populations: In particular, discuss generalizability to a general Internet population, outside of a RCT setting, and general patient population, including applicability of the study results for other organizations

	1	2	3	4	5	
subitem not at all important	○	○	○	○	◉	essential

Borrar selección

Does your paper address subitem 21-i?

Copy and paste relevant sections from the manuscript (include quotes in quotation marks "like this" to indicate direct quotes from your manuscript), or elaborate on this item by providing additional information not in the ms, or briefly explain why the item is not applicable/relevant for your study

Not applicable.

Estás editando tu respuesta. Si compartes esta URL, otros usuarios también podrán editar la respuesta.

RELLENAR UNA RESPUESTA NUEVA

21-ii) Discuss if there were elements in the RCT that would be different in a routine application setting

Discuss if there were elements in the RCT that would be different in a routine application setting (e.g., prompts/reminders, more human involvement, training sessions or other co-interventions) and what impact the omission of these elements could have on use, adoption, or outcomes if the intervention is applied outside of a RCT setting.

	1	2	3	4	5	
subitem not at all important	○	○	○	○	◉	essential

Borrar selección

Does your paper address subitem 21-ii?

Copy and paste relevant sections from the manuscript (include quotes in quotation marks "like this" to indicate direct quotes from your manuscript), or elaborate on this item by providing additional information not in the ms, or briefly explain why the item is not applicable/relevant for your study

Not applicable.

OTHER INFORMATION

23) Registration number and name of trial registry

Does your paper address CONSORT subitem 23? *

Copy and paste relevant sections from the manuscript (include quotes in quotation marks "like this" to indicate direct quotes from your manuscript), or elaborate on this item by providing additional information not in the ms, or briefly explain why the item is not applicable/relevant for your study

The trial was registered at ClinicalTrials.gov (NCT04649736).

Estás editando tu respuesta. Si compartes esta URL, otros usuarios también podrán editar la respuesta. RELLENAR UNA RESPUESTA NUEVA

24) Where the full trial protocol can be accessed, if available

Does your paper address CONSORT subitem 24? *

Cite a Multimedia Appendix, other reference, or copy and paste relevant sections from the manuscript (include quotes in quotation marks "like this" to indicate direct quotes from your manuscript), or elaborate on this item by providing additional information not in the ms, or briefly explain why the item is not applicable/relevant for your study

The full trial protocol is not openly accessible to the general public.

25) Sources of funding and other support (such as supply of drugs), role of funders

Does your paper address CONSORT subitem 25? *

Copy and paste relevant sections from the manuscript (include quotes in quotation marks "like this" to indicate direct quotes from your manuscript), or elaborate on this item by providing additional information not in the ms, or briefly explain why the item is not applicable/relevant for your study

"This study was funded by the Peruvian National Council of Science Technology and Technology Innovation (CONCYTEC - PROCIENCIA). Lima, Peru (Contract #077-2020-FONDECYT). ANS, SMH, AGL and WC are also funded by 5D43TW011502-02 Fogarty International Centre of the U.S. National Institutes of Health Chronic Pulmonary Disease D43 Training Grant."

X27) Conflicts of Interest (not a CONSORT item)

Estás editando tu respuesta. Si compartes esta URL, otros usuarios también podrán editar la respuesta.

RELLENAR UNA RESPUESTA NUEVA

X27-i) State the relation of the study team towards the system being evaluated

In addition to the usual declaration of interests (financial or otherwise), also state the relation of the study team towards the system being evaluated, i.e., state if the authors/evaluators are distinct from or identical with the developers/sponsors of the intervention.

	1	2	3	4	5	
subitem not at all important	○	○	○	○	◉	essential

Borrar selección

Does your paper address subitem X27-i?

Copy and paste relevant sections from the manuscript (include quotes in quotation marks "like this" to indicate direct quotes from your manuscript), or elaborate on this item by providing additional information not in the ms, or briefly explain why the item is not applicable/relevant for your study

Not applicable.

About the CONSORT EHEALTH checklist

As a result of using this checklist, did you make changes in your manuscript? *

◉ yes, major changes

○ yes, minor changes

○ no

What were the most important changes you made as a result of using this checklist?

Estás editando tu respuesta. Si compartes esta URL, otros usuarios también podrán editar la respuesta.

RELLENAR UNA RESPUESTA NUEVA

How much time did you spend on going through the checklist INCLUDING making changes in your manuscript *

3 hours

As a result of using this checklist, do you think your manuscript has improved? *

- (x) yes
- ( ) no
- ( ) Otro:

Would you like to become involved in the CONSORT EHEALTH group?

This would involve for example becoming involved in participating in a workshop and writing an "Explanation and Elaboration" document

- (x) yes
- ( ) no
- ( ) Otro:

Borrar selección

Any other comments or questions on CONSORT EHEALTH

Tu respuesta

Estás editando tu respuesta. Si compartes esta URL, otros usuarios también podrán editar la respuesta.

RELLENAR UNA RESPUESTA NUEVA

STOP - Save this form as PDF before you click submit

To generate a record that you filled in this form, we recommend to generate a PDF of this page (on a Mac, simply select "print" and then select "print as PDF") before you submit it.

When you submit your (revised) paper to JMIR, please upload the PDF as supplementary file.

Don't worry if some text in the textboxes is cut off, as we still have the complete information in our database. Thank you!

Final step: Click submit !

Click submit so we have your answers in our database!

Enviar

Nunca envíes contraseñas a través de Formularios de Google.

Este contenido no ha sido creado ni aprobado por Google. Notificar uso inadecuado - Términos del Servicio - Política de Privacidad

Google Formularios

Estás editando tu respuesta. Si compartes esta URL, otros usuarios también podrán editar la respuesta. RELLENAR UNA RESPUESTA NUEVA
